# FOXP4-mediated induction of PTK7 activates the Wnt/β-catenin pathway and promotes ovarian cancer development

**DOI:** 10.1038/s41419-024-06713-7

**Published:** 2024-05-13

**Authors:** Jing Ji, Qilan Qian, Wenhao Cheng, Xiaoqing Ye, Aixin Jing, Shaojie Ma, Yuanyuan Ding, Xinhui Ma, Yasong Wang, Qian Sun, Xiujun Wang, Yulu Chen, Lan Zhu, Qing Yuan, Menghan Xu, Jingting Qin, Lin Ma, Jiayan Yang, Meiqi Zhang, Ting Geng, Sen Wang, Dan Wang, Yizhuo Song, Boyu Zhang, Yuting Xu, Linyu Xu, Shunfang Liu, Wei Liu, Bin Liu

**Affiliations:** 1 Jiangsu Key Laboratory of Marine Pharmaceutical Compound Screening, College of Pharmacy, Jiangsu Ocean University, 222005 Lianyungang, China; 2 Cancer Center and Department of Pharmacology and Toxicology, Medical College of Wisconsin, Milwaukee, WI 53226 USA; 3 The First People’s Hospital of Lianyungang, the First Affiliated Hospital of Kangda College of Nanjing Medical University, 7 Zhenhua Road, Haizhou, 222061 Lianyungang, Jiangsu PR China; 4 Department of Oncology, Tongji Hospital, Tongji Medical College, Huazhong University of Science and Technology, 430030 Wuhan, China

**Keywords:** Ovarian cancer, Tumour biomarkers

## Abstract

Ovarian cancer (OV) poses a significant challenge in clinical settings due to its difficulty in early diagnosis and treatment resistance. FOXP4, belonging to the FOXP subfamily, plays a pivotal role in various biological processes including cancer, cell cycle regulation, and embryonic development. However, the specific role and importance of FOXP4 in OV have remained unclear. Our research showed that FOXP4 is highly expressed in OV tissues, with its elevated levels correlating with poor prognosis. We further explored FOXP4’s function through RNA sequencing and functional analysis in FOXP4-deficient cells, revealing its critical role in activating the Wnt signaling pathway. This activation exacerbates the malignant phenotype in OV. Mechanistically, FOXP4 directly induces the expression of protein tyrosine kinase 7 (PTK7), a Wnt-binding receptor tyrosine pseudokinase, which causes abnormal activation of the Wnt signaling pathway. Disrupting the FOXP4-Wnt feedback loop by inactivating the Wnt signaling pathway or reducing FOXP4 expression resulted in the reduction of the malignant phenotype of OV cells, while restoring PTK7 expression reversed this effect. In conclusion, our findings underscore the significance of the FOXP4-induced Wnt pathway activation in OV, suggesting the therapeutic potential of targeting this pathway in OV treatment.

## Introduction

Ovarian cancer, a malignancy that poses a substantial threat to women globally, remains a formidable challenge in oncology. Despite constituting only 2.5% of all female malignancies, ovarian cancer contributes to 5% of cancer-related fatalities among women, highlighting its grave impact [1, 2]. The majority of ovarian cancer cases are diagnosed at advanced stages, typically afflicting older individuals, resulting in dismal survival rates. A primary hurdle in combating this disease lies in the complexities of early detection. The absence of reliable screening modalities, coupled with nonspecific symptoms often mimicking benign conditions, severely impedes early intervention efforts [3]. Conventional treatment modalities for ovarian cancer, encompassing radiotherapy, chemotherapy, and surgery, entail considerable physiological tolls on patients [4]. Therefore, comprehending the molecular intricacies underpinning ovarian cancer pathogenesis is imperative for devising more efficacious and less deleterious therapeutic interventions, with the ultimate goal of enhancing patient outcomes.

In Drosophila melanogaster, the fork head box (FOX) protein is classified within the “helix-loop-helix” protein group [5]. Among the FOXP subfamily, FOXP4 stands out, with research highlighting its significant involvement in embryonic development, cell cycle modulation, and tumorigenesis [6]. Interactions between FOXP proteins and various signaling pathways underscore the critical role of FOXP4 in the pathogenesis of hepatocellular carcinoma [7], breast cancer [8], medulloblastoma [9], and osteosarcoma [10]. However, the dual nature of FOXP proteins as either oncogenes or tumor suppressors varies across different cancer types. While FOXP4 functions as a tumor suppressor in kidney cancer, it acts as an oncogene in hepatocellular carcinoma [11]. The precise conditions or factors governing these opposing effects of FOXP4 remain elusive. Deciphering the molecular mechanisms underlying FOXP4’s function holds promise for identifying novel therapeutic targets against cancer, thus necessitating a thorough exploration of FOXP4’s role and its correlation with ovarian cancer.

Protein tyrosine kinase 7 (PTK7) belongs to the pseudokinase subgroup of receptor tyrosine kinases, playing pivotal roles across diverse biological processes [12]. Its functions extend to cell development [13], planar cell polarity (PCP) [14], embryonic development [15], neural tube closure [16], and heart development [17]. PTK7 amplifies the activation of FGFR1 and EGFR, crucial in transmitting oncogenic signals that foster cancer initiation and metastasis [18]. Moreover, PTK7 engages with Wnt ligands like Wnt3a and Wnt5a, thereby activating non-canonical Wnt signaling pathways. Additionally, it participates in canonical Wnt/β-catenin signaling, implicated in various pathological conditions, encompassing embryonic anomalies, neurodegenerative disorders, and cancer [19–21]. While the role of PTK7 in cancer progression garners increasing recognition [22], elucidating the precise molecular mechanisms governing PTK7’s impact on ovarian tumor advancement and its regulatory factors necessitates meticulous investigation.

This study has pinpointed FOXP4 as a pivotal signaling factor impacting ovarian cancer (OV) tumor progression. We delineated a signaling cascade implicating Wnt/β-catenin/TCF4-FOXP4-PTK7. FOXP4 functions as a transcriptional target of Wnt/β-catenin/TCF4, facilitating PTK7 transcription and thereby fostering OV tumor development and advancement. These findings underscore FOXP4’s potential as a prognostic biomarker for ovarian cancer and unveil novel therapeutic avenues, charting promising trajectories for future ovarian cancer investigations.

## Material and methods

### Data collection and analysis

This study utilized the GEPIA database (http://gepia2.cancer-pku.cn) to retrieve the differential expression of FOXP4 in OV, and compare it with normal tissue. Additionally, the microarray dataset GSE140082 (Platform: GPL14951) downloaded from the NCBI website and analyzed using R software, examined FOXP4 expression across different OV TNM stages. The relationship between FOXP4 mRNA levels and clinical pathological features was explored using the UALCAN online platform (https://ualcan.path.uab.edu). Protein levels of FOXP4 in both normal and OV tissues were assessed using data from the Clinical Proteomic Tumor Analysis Consortium (CPTAC) database. Immunohistochemical staining images from the Human Protein Atlas (HPA) database(https://www.proteinatlas.org/) highlighted the presence of FOXP4 in ovarian cancer cells. Survival analysis, including overall survival (OS) and progression-free survival (PFS), was performed with Kaplan-Meier online software (http://kmplot.com/) to evaluate the impact of FOXP4 expression levels in ovarian cancer patients. Gene expression data from The Cancer Genome Atlas (TCGA) were used to explore the association between FOXP4 and its related genes at the mRNA level in ovarian cancer cell lines. To forecast TCF4 binding sites in the human FOXP4 promoter and potential FOXP4 binding sites in the PTK7 promoter, the human FOXP4 and PTK7 gene promoter regions from EPD (https://epd.epfl.ch/index.php) were added to the JASPAR website (https://jaspar.genereg.net/). Data visualization was achieved through the “pheatmap” and “ggplot2” packages in RStudio, illustrating cluster and volcano plots, respectively. Kyoto Encyclopedia of Genes and Genomes (KEGG) analysis was performed on the downregulated gene set after FOXP4 knockout using the Enrich KEGG function of the RStudio downloadable software package “clusterProfiler.” Gene Set Enrichment Analysis (GSEA) software (http://www.gsea-msigdb.org/) and the TCGA ovarian cancer dataset were used to analyze the signaling pathways regulated by FOXP4 in ovarian cancer.

### RNA interference, RNA isolation and real-time polymerase chain reaction (PCR)

The human lentiviral expressing short hairpin RNA (shRNA) plasmids were utilized from Sigma-Aldrich. The design target sequences for the shRNA plasmids in present study were as follows: FOXP4-shRNA1 (CCAGGGAACAATGACAGCAAA), FOXP4-shRNA2 (CCAGTTTATCAAACACCTCAA), FOXP4-shRNA3 (GTTCGCCTATTTCCGCAGAAA), FOXP4-shRNA4 (CCAGAATCATGAGTTCTACAA), FOXP4-shRNA5 (CACCAGGATGTTCGCCTATTT), PTK7-shRNA (CCTGAGGATTTCCAAGAGCAA), and β-catenin-shRNA (TTGTTATCAGAGGACTAAATA). OV (A2780 and OVCAR8) cells were infected with lentiviral plasmids (FOXP4-shRNA, PTK7-shRNA, and β-catenin-shRNA) and subsequently subjected to selection using varying concentrations of puromycin over two weeks. Total RNA was then extracted from the treated cells using TRIzol reagent (Invitrogen, USA) and reverse transcribed into cDNA with a kit from Promega. The resulting cDNA underwent RT-PCR using specific cycle settings: initial denaturation at 95°C for 30 s followed by 40 cycles at 95°C for 5 s and 62°C for 30 s. Melting curve analysis was performed concurrently to assess the relative expression levels of the target genes, using GAPDH as a normalization control for the Ct values. The primer sequences used were as follows: CCND1 forward, 5′-GCTGCGAAGTGYGAAACCATC-3′; reverse, 5′-CCTCCTTCTGCACACATTTGAA-3′; GSK3B forward, 5′-GGCAGCATGAAAGTTAGCAGA-3′; reverse, 5′-GGCGACCAGTTCTCCTGAATC-3′; β-catenin forward, 5′-CATCTACACAGTTTGATGCTGCT-3′; reverse, 5′-GCAGTTTTGTCAGTTCAGGGA-3’; c-Myc forward, 5′-GGCTCCTGGCAAAAGGTCA-3′; reverse, 5′-CTGCGTAGTTGTGCTGATGT-3′; FOXP4 forward, 5′-GACAGCCTACTGTGCTCACAT-3′; reverse, 5′- TTGCACTCTCCGTGTCCGTA-3′; PTK7 forward, 5′-ACACTTCGTTGCCACATTGAT-3′; reverse, 5′-CAGCAGGAATACAGCCCAC-3′; GAPDH forward, 5′- GGAGCGAGATCCCTCCAAAAT-3′; reverse, 5′-GGCTGTTGTCATACTTCTCATGG-3′. MKI67 forward, 5′-ACGCCTGGTTACTATCAAAAGG-3′; reverse, 5′-CAGACCCATTTACTTGTGTTGGA -3′. FXOP1 forward, 5′-TCCCGTGTCAGTGGCTATGAT-3′; reverse, 5′-CTCTTTAGGCTGTTTTCCAGCAT-3′.

### Cell culture and clinical tissue samples

The 293T and human ovarian cancer cell lines A2780 and OVCAR8, provided by the American Type Culture Collection (ATCC), were confirmed to be free of Mycoplasma infection. Cells were obtained by adding 10% fetal bovine serum (FBS) in DMEM or 1640 medium (Invitrogen, CA, USA) and cultured at 37°C and 5% CO_2_. The media also included 100 mg/mL of streptomycin and 100 units/mL of penicillin to prevent bacterial contamination. Chemical reagents LiCl and ICG-001 were obtained from Selleck, while DMSO was purchased from Sigma-Aldrich. Tumor and normal ovarian tissues were provided by the First People’s Hospital of Lianyungang City. All samples were collected with the informed consent of participants, patients, and their families and approved by the Medical Ethics Committee of the First People’s Hospital of Lianyungang City.

### Plasmids and transfection

Gene segments of FOXP4 and PTK7 were inserted into the pcDNA3.1-HA and pbabe-FLAG vectors, respectively. As previously mentioned, truncated versions of PTK7 and FOXP4 were subcloned into the pbabe-FLAG vector. HEK293T cells, utilized as tool cells, were co-transfected with 1 μg of the target plasmid, the viral packaging plasmid, and Lipofectamine 2000 (Invitrogen) after being starved for 1 h. The medium was replaced after 8 h, and the virus was harvested following 48 h of incubation. OV cell lines (A2780 and OVCAR8) were infected with lentiviral particles and polybrene (Beyotime). After 48 h of infection, the desired cell lines were obtained by screening using puromycin.

### RNA Sequencing

The virus of FOXP4-shRNA was prepared in tool cell 293T and transduced into new 293T cells, followed by puromycin selection to obtain FOXP4-KO cell lines. The cells were processed using TRIzol reagent (Invitrogen Company, USA) to extract RNA, which was then sent to Shanghai Sangon Technology Co., LTD for RNA sequencing (RNA-Seq). The sequencing data obtained were subsequently analyzed using bioinformatics techniques to derive meaningful insights. The sequencing data were visualized using R software. The RNA-Seq data supporting this publication are available in the Gene Expression Omnibus repository under the accession number GSE242332.

### Immunoblotting analysis

Cell samples were centrifuged to remove the supernatant. Total protein was extracted by lysing the cells with an appropriate amount of 1× loading buffer (P0015A, Beyotime). Proteins were separated by 12% SDS-PAGE and transferred onto a polyvinylidene difluoride (PVDF) membrane. The membrane was blocked using 5% skim milk for 45 min before being washed three times with PBST, each wash lasting 5 min. It was then incubated overnight with primary antibodies at 4°C. After incubation, the membrane was placed on a shaker (75 rpm) and washed with PBST, followed by 45 min of incubation with secondary antibodies at room temperature. After another round of PBST washing, chemiluminescent detection was performed. The following primary antibodies were used: anti-β-catenin (Santa Cruz, sc-7963), anti-FOXP4 (Santa Cruz, sc-390892), anti-PTK7 (Bethyl Laboratories, A304-451A), anti-Cyclin D1 (Abcam, ab134175), anti-GSK3B (Abcam, ab76225), anti-c-Myc (Abcam, ab32072), anti-E-cadherin (Abcam, ab40772), anti-N-cadherin (Abcam, AB207608), anti-Vimentin (Abcam, ab92547), and anti-β-actin (Santa Cruz, sc-8432).

### Luciferase, and Chip assay

The TCF/LEF1 luciferase reporters were purchased from Yeasen. Whole genomic DNA was extracted from A2780 cells, and the promoter regions of the FOXP4 and PTK7 genes were amplified and subsequently cloned into the pGL4.15 vector. A dual luciferase assay approach was used to assess intracellular luciferase activity after 293 T cells were transfected in 24-well plates. Firefly luciferase activity was normalized to Renilla luciferase activity to enhance transfection efficiency. Chromatin immunoprecipitation (ChIP) assays were conducted using a ChIP assay kit. Cells were fixed with cold formaldehyde and then sonicated to shear the DNA into fragments. Using antibodies against FOXP4 and PTK7 or normal serum IgG as negative controls, the supernatants were incubated overnight at 4°C. Using a DNA purification kit, the precipitated complex was purified and eluted, and the resulting pure DNA was utilized as a PCR template. The GAPDH promoter served as an additional negative control in these experiments.

### Caspase-3/7 activity assay

The CellEvent™ Caspase-3/7 Green ReadyProbes™ Reagent (Thermo Fisher C10427) protocol was adhered to for apoptosis detection. A2780 cells transfected with PLKO, shFOXP4#1, and shFOXP4#2 were seeded in equal amounts into 96-well plates. The fluorescence intensity of the caspase-3/7 activity was measured using a Synergy H1 microplate reader at excitation and emission wavelengths of 502 nm and 530 nm, respectively. The fluorescence data were normalized against a control consisting only of the culture medium to account for background fluorescence.

### Cell proliferation and colony formation assay

The viability of the cells was assessed using the BrdU assay and a cell growth curve experiment. In the cell growth curve assay, logarithmically growing cells were harvested using 0.25% trypsin solution (Thermo Fisher 25200056) and resuspended in DMEM medium. Cell suspensions of PLKO, shFOXP4#1, and shFOXP4#2 were seeded in 24-well plates at densities ranging from 2 × 10^4^ to 5 × 10^4^ cells/mL. Cell counts were recorded at 0, 24, 48, and 72 h post-seeding by trypsinizing and observing under a microscope to construct the growth curves. For the BrdU assay, 96-well plates were seeded with equal numbers of A2780 and OVCAR8 cells expressing PLKO, shFOXP4#1, and shFOXP4#2, following the instructions of the BrdU Cell Proliferation Assay Kit (Cell Signaling). Proliferation was quantified by measuring absorbance at designated wavelengths. In the colony formation assay, 1×10^4^ cells were seeded in 6-well plates. PLKO, shFOXP4#1, and shFOXP4#2 cells in A2780; empty vector (EV) + DMSO, EV + ICG-001, FOXP4 + DMSO, FOXP4 + ICG-001; PLKO, shFOXP4#1, shFOXP4#1 + PTK7; EV, FOXP4, FOXP4+sh-PTK7; as well as PLKO, shFOXP4#1, and shFOXP4#2 cells in OVCAR8 were incubated in a CO_2_ incubator at 37 °C for 7–14 days until colonies grow to the appropriate size. The colonies were fixed with cryo-pretreated 4% paraformaldehyde, stained with crystal violet, and those containing more than 50 cells were counted to evaluate cloning capacity.

### Cell migration and invasion assay

A2780 cells grown in a 6-well plate were cultured to reach 80% confluency over 24 h. For the migration assay, these cells were then treated according to the experimental protocols, and an appropriate volume of cell suspension was added to the upper chamber of a transwell apparatus, while the lower chamber was filled with medium conducive to normal cell growth. The cells were incubated for 24 h, after which they were fixed with 4% paraformaldehyde and stained with crystal violet. Quantitative analysis of the migrated cells was conducted using ImageJ software. For the invasion assay, the upper chamber of the transwell was first coated with melted matrigel, which was allowed to solidify for 1 h in the incubator. The lower chamber was filled with the required medium for normal cell growth, and cells including PLKO, shFOXP4#1, and shFOXP4#2 A2780, as well as other experimental groups from A2780 and OVCAR8 cells as outlined in supplementary fig. 3, were seeded in the upper chamber. After 48 h, the cells were morphologically fixed with 4% paraformaldehyde and subsequently stained for easy visualization. The number of invaded cells was counted in five randomly selected fields to average the results, and this data was used to analyze cell migration and invasion capabilities.

### Xenograft tumors

The National Rodent Laboratory Animal Resources in Shanghai, China provided BALB/c nude mice, which were housed in designated facilities. Initially, two groups consisting of six mice each were formed via random selection. Each mouse group received subcutaneous injections of A2780 cells (1×10^7^ cells) suspended in 50 µL of DMEM medium. The cells were transfected with either Con-shRNA, FOXP4-shRNA#1, or FOXP4-shRNA#2. After four weeks, the tumors were excised, weighed, and photographed. Subsequently, the mice were re-assigned into three new groups, each containing six mice, and received subcutaneous injections on both sides with A2780 cells (1×10^7^ cells) transfected with either shFOXP4#1, shFOXP4#1 + PTK7, or pLKO. Tumor growth and volume were monitored weekly, and the tumors were photographed at the study’s conclusion. Similarly, three groups of mice were randomly assigned, for a total of 18 mice. A2780 cells (1×10^7^ cells) containing EV, FOXP4, and FOXP4+Sh-PTK7 were injected subcutaneously into both sides of the mice. Tumor volume and growth were monitored weekly. Finally, the tumors were photographed. All experimental protocols involving animals were approved by the Jiangsu Ocean University Ethics Committee.

### Statistical analyses

Statistical analyses were performed using GraphPad Prism 8.0 software. Differences between two groups were compared using the Student’s *t* test or one-way analysis of variance (ANOVA) with Tukey’s test for post hoc comparisons. Comparisons among multiple groups were analyzed using a one-way ANOVA. The levels of statistical significance were denoted as *p < 0.05, **p < 0.01, and ***p < 0.001. A *p*-value less than 0.05 was considered statistically significant.

## Result

### Upregulation of FOXP4 is associated with poor prognosis in ovarian cancer patients

**Fig. 1:**
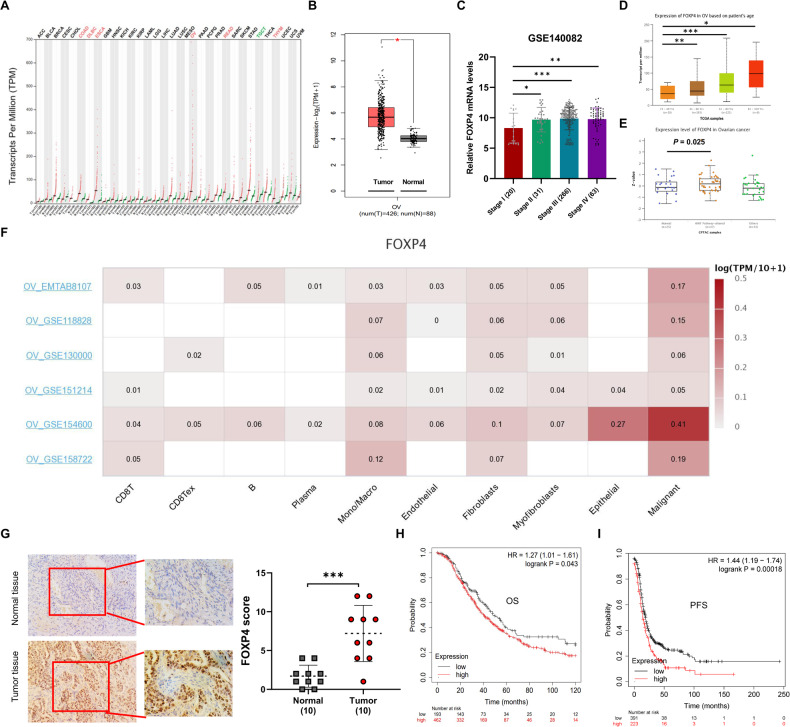
Upregulation of FOXP4 correlates with poor prognosis for OV patients. **A** The expressions of FOXP4 in 33 types of human cancer in data from TCGA dataset through GEPIA2 online website (https://gepia2.cancer-pku.cn). The gene expression profile across all tumor samples and paired normal tissues is shown as a dot plot. Ret dot represents cancer sample and green dot represents normal sample. **B** Boxplot analysis of differences in FOXP4 expression levels in ovarian tissues from the GEPIA2 website. T, Tumor tissues; N, Normal tissues; *P-value was set at 0.01. **C** The mRNA expression of FOXP4 from different OV TNM stages in an independent OV patient cohort (GSE140082). *p < 0.05, **p < 0.01, ***p < 0.001. **D** Using UALCAN network platform (http://ualcan.path.uab.edu/) analysis FOXP4 mRNA expression level and patient age. This is visually represented by a box plot. *p < 0.05, **p < 0.01, ***p < 0.001. **E** The protein level of FOXP4 from normal ovary tissue and OV tissues from Clinical Proteomic Tumor Analysis Consortium (CPTAC). FOXP4 was selectively highly expressed in Wnt pathway-altered OV tissues. **F** Single-cell sequencing showed that FOXP4 is highly expressed in OV malignant tumor cells. **G** The representative IHC staining of FOXP4 protein in OV tissue (Ovarian serous cystadenocarcinoma) and normal tissue (ovary) from the Human Protein Atlas Project (http://www.proteinatlas.org/). ***p < 0.001. **H**, **I** The prognostic values of FOXP4 in OV patients. The survival curves comparing OV patients with high (red) and low (black) FOXP4 expression levels were plotted using the online Kaplan–Meier Plotter (https://kmplot.com/). OS, overall survival rate (**H**); PFS, progression free survival rate (**I**).

Utilizing TCGA and GTEx datasets, we analyzed FOXP4 mRNA expression across 33 human cancers and corresponding normal tissues using the GEPIA tool (Fig. 1A). FOXP4 was selectively upregulated in 6 cancer types when compared to their respective normal tissues. From the GTEx dataset revealed significantly higher FOXP4 mRNA expression in 426 OV tissues compared to 88 normal ovarian tissues (Fig. 1B). An independent cohort (GSE140082) further validated this observation, with late-stage OV patients exhibiting higher FOXP4 mRNA expression than early-stage patients (Fig. 1C). The relationship between FOXP4 mRNA expression and clinical pathological characteristics in OV samples was investigated using the UALCAN tool. We observed an increase in FOXP4 mRNA expression levels with patient age (Fig. 1D). Analysis of FOXP4 protein levels in OV tissues revealed selective overexpression in OV tissues with altered Wnt pathway (Fig. 1E). In multiple single-cell sequencing datasets of ovarian cancer, we found that FOXP4 was predominantly expressed in malignant tumor cells, while it was expressed at lower levels in stromal cells and immune cells (Fig. 1F). Immunohistochemical staining data from the HPA database confirmed FOXP4’s differential protein expression, showing higher levels in OV than in normal ovarian tissues (Supplementary Fig. 1A). Furthermore, immunohistochemical analysis showed significantly higher FOXP4 protein content in OV tissues than in adjacent normal ovarian tissues (Fig. 1G). The clinical significance of FOXP4 was assessed by examining the correlation between the FOXP4 transcript levels and the survival outcomes of ovarian cancer patients with the aid of the Kaplan-Meier Plotter tool. The results showed that patients with higher FOXP4 expression levels had lower overall survival (OS) (Fig. 1H) and progression-free survival (PFS) (Fig. 1I), while post-progression survival (PPS) did not show a significant decrease (Supplementary Fig. 1B). These findings suggest that FOXP4 is significantly upregulated in ovarian cancer and that high FOXP4 levels are associated with poor prognosis in OV patients.

### FOXP4 is a transcriptional target of the Wnt/β-catenin/TCF4 signaling pathway

**Fig. 2:**
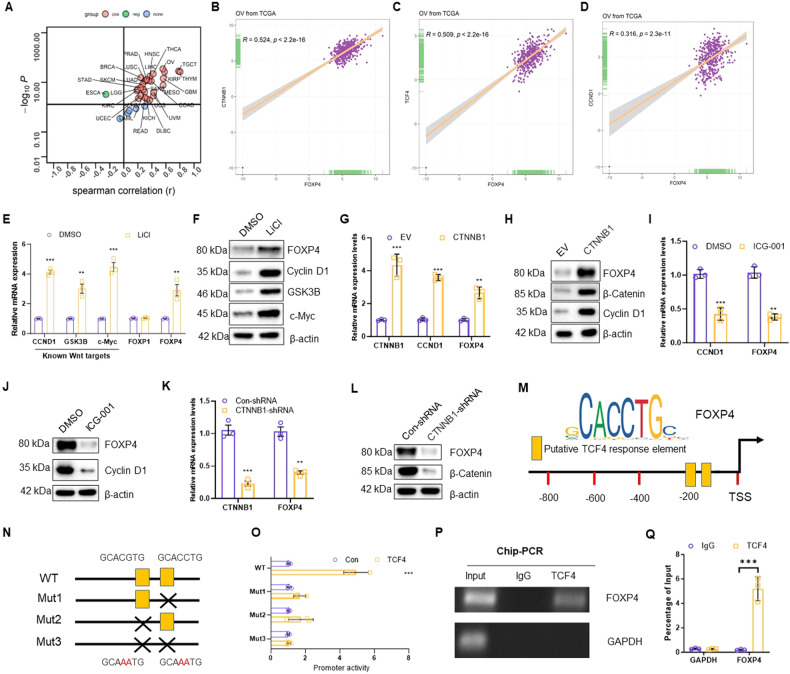
FOXP4 is a transcriptional target for the Wnt/β-catenin/TCF4 signaling. **A** The spearman correlation between the expression of FOXP4 and the HALLMARK_WNT_BETA_CATENIN_SIGNALING in all tumor types in TCGA. Red represents positive expression and green represents negative expression. **B** The mRNA correlation between FOXP4 and CTNNB1 in OV from TCGA database. **C** The mRNA correlation between FOXP4 and TCF4 in OV from TCGA database. **D** The mRNA correlation between FOXP4 and CCND1 in OV from TCGA database. **E** The mRNA expression levels of FOXP4, FOXP1 and three known downstream target genes of Wnt/β-catenin (CCND1, GSK3B, and c-Myc) in A2780 cells treated with DMSO or LiCl were determined by real-time PCR, *n* = 3. **p < 0.01 vs DMSO, ***p < 0.001 vs DMSO. **F** Immunoblot analysis was used to detect the protein expression levels of FOXP4 and three known Wnt/β-catenin downstream target genes CCND1, GSK3B, and c-Myc in A2780 cells treated with DMSO or LiCl. **G** Real-time PCR was used to measure mRNA levels of β-catenin, CCND1, and FOXP4 after transfection with EV or CTNNB1 in A2780 cells, *n* = 3. **p < 0.01 vs EV, ***p < 0.001 vs EV. **H** Immunoblot analysis was conducted to assess the protein expression of β-catenin, CCND1, and FOXP4 in A2780 cells following transfection with EV or β-catenin. **I** Real-time PCR was used to quantify mRNA levels of CCND1 and FOXP4 in OVCAR8 cells treated with either DMSO or ICG-001, *n* = 3. ***p < 0.001 vs DMSO. **J** Immunoblot analysis was used to determine the protein expression levels of CCND1 and FOXP4 in OVCAR8 cells after treatment with either DMSO or ICG-001. **K** Real-time PCR was utilized to quantify the mRNA expression of CTNNB1 and FOXP4 in OVCAR8 cells transfected with Con-shRNA or CTNNB1-shRNA, *n* = 3. **p < 0.01 vs Con-shRNA, ***p < 0.001 vs Con-shRNA. **L** Immunoblot analysis was conducted to determine the protein levels of β-catenin and FOXP4 in OVCAR8 cells transfected with Con-shRNA or CTNNB1-shRNA. **M** Schematic diam shows human FOXP4 gene promoter and two putative TCF4 binding sites. TSS: transcription start site. **N** The human FOXP4 promoter contains two TCF4 response elements. Point mutation was highlighted with black cross. **O** TCF4 was co-transfected with indicated plasmids into 293T cells for 36 h. The luciferase activity was then measured, *n* = 3. ***p < 0.001 vs Con. **P**, **Q**. A2780 cells stably expressing TCF4 were subjected to Chip-PCR detection, the human GAPDH promoter served as a negative control, *n* = 3. ***p < 0.001 vs IgG.

To elucidate the upstream molecular mechanisms and pathways involved in FOXP4 transcriptional regulation, we first extracted FOXP4-related signaling pathways from the TCGA database. Analysis revealed a strong association between FOXP4 expression and Wnt/β-catenin signaling pathway activation in the TCGA database (Fig. 2A). Furthermore, FOXP4 showed a strong correlation with several Wnt pathway-related genes, including CTNNB1, TCF4, and CCND1 in OV (Fig. 2B–D), suggesting that FOXP4 may be involved in this pathway as a potential target gene. To validate this, OV cells were treated with LiCl, a known Wnt pathway activator, which induced high levels of FOXP4 transcription and expression alongside Wnt pathway activation (Fig. 2E, F). Additionally, overexpression of β-catenin in OV cells not only induced the known target gene CCND1 but also FOXP4 (Fig. 2G–H). Conversely, treatment with the potent Wnt/β-catenin inhibitor ICG-001 or silencing of CTNNB1 expression significantly suppressed the expression of CCND1 and FOXP4 in OV cells (Fig. 2I–L). Thus, these results suggest that FOXP4 may participate in the Wnt signaling pathway and exert effects as a downstream target. Additionally, through a search in the JASPAR online database (http://jaspar.genereg.net/), Two potential TCF4 response elements were found in the FOXP4 promoter region (Fig. 2M), showing that FOXP4 could be β-catenin/TCF4’s transcriptional target. To confirm this possibility, different luciferase reporter constructs for FOXP4 incorporating the two putative TCF4 response elements were generated. As expected, TCF4 effectively enhanced the activity of the promoter when both elements were present, but lost the ability to activate the promoter when both elements were mutated. It suggests that two transcriptions start site (TSS) elements are extremely important for the activation of the TCF4-dependent promoter (Fig. 2N, O). Chip validation revealed a certain binding between TCF4 and FOXP4 (Fig. 2P, Q, Supplementary Fig. 2A, B). Taken together, the Wnt/β-catenin/TCF4 signaling pathway may have a new transcriptional target, FOXP4.

### Development of OV malignant tumor cells require FOXP4

**Fig. 3:**
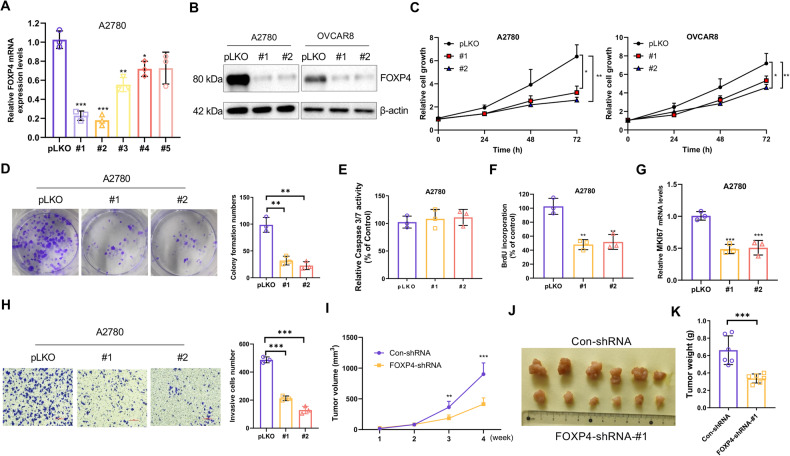
FOXP4 is required for OV cells to maintain malignancy. **A** Real-time PCR was used to measure FOXP4 mRNA in A2780 cells transfected with pLKO or five specific FOXP4 shRNAs, *n* = 3. *p < 0.05 vs pLKO, **p < 0.01 vs pLKO, ***p < 0.001 vs pLKO. **B** The OV (A2780 and OVCAR8) cells stably expressing pLKO or two specific shRNAs against FOXP4 were subjected to immunoblotting with indicated antibodies. **C** The cell growth curve of A2780 and OVCAR8 cells in (**B**), *n* = 3. *p < 0.05 vs pLKO, **p < 0.01 vs pLKO. **D** Clonogenic assay of A2780 cells in (**B**), *n* = 3. *p < 0.05 vs pLKO, **p < 0.01 vs pLKO. **E** Caspase3/7 activity was measured in A2780 cells in (**B**). The *y*-axis indicates the Caspase3/7 activity over cell number. The value given for the caspase activity in control-infected cells was set as 100. **F** Incorporation of BrdU in A2780 cells in (**B**) was measured by ELISA, *n* = 3. **p < 0.01 vs pLKO. **G** Real-time PCR analysis of MKI67 mRNA in cells from (**B**), *n* = 3. ***p < 0.001 vs pLKO. **H** Migration rates of cells from (**B**) were evaluated, *n* = 3. **p < 0.01 vs pLKO, ***p < 0.001 vs pLKO. **I** A2780 cells stably expressing Con-shRNA or FOXP4-shRNA were inoculated subcutaneously in nude mice, respectively. Tumor volume was monitored over time, *n* = 6. **p < 0.01 vs Con-shRNA, ***p < 0.001 vs Con-shRNA. **J** Image representing the tumor on day 28, *n* = 6. **K** Bar graphs represent the weight of the tumor on day 28, *n* = 6. ***p < 0.001 vs Con-shRNA.

Given these above observations, we deduced FOXP4’s involvement in ovarian carcinogenesis and proceeded to explore its biological role in OV. Firstly, A2780 and OVCAR8 cells were infected with lentiviral particles harboring shRNA targeting the control region or five different regions of FOXP4. Knockdown efficiency was assessed via real-time PCR (Fig. 3A), leading to the selection of the two most potent shRNAs for establishing stable FOXP4-silenced cell lines in A2780 and OVCAR8 cells. FOXP4 knockdown was additionally confirmed through immunoblotting (Fig. 3B). Analysis the growth curves and colony formation revealed that complete elimination of FOXP4 remarkably inhibited cell growth and reduced the clonogenic capacity of cells (Fig. 3C, D, Supplementary Fig. 3A). Given the minimal changes in Caspase 3/7 activity (Fig. 3E, Supplementary Fig. 3B), it indicated that silencing of FOXP4 did not result in apparent apoptosis in OV cells. Notably, FOXP4 silencing led to a decrease in both bromodeoxyuridine (BrdU)-binding cells and mRNA levels of the proliferation marker MKI67 (Fig. 3F, G, Supplementary Fig. 3C). Hence, these findings suggest that FOXP4 is crucial for the regulation of OV cell proliferation. Next, we expanded our investigation of FOXP4’s biological function to include tumor cells’ capacity for migration and invasion. Transwell assays demonstrated a significant reduction in OV cell migration and invasion upon FOXP4 knockout (Fig. 3H, Supplementary Fig. 3D), underscoring FOXP4’s critical role in sustaining OV cells in vitro. OV cells were injected subcutaneously in nude mice. Tumor size and weight were significantly reduced after FOXP4 silencing compared with the control (Fig. 3I–K). Together, these results indicate that the involvement of FOXP4 in OV tumor development promotion.

### FOXP4 functions as a positive regulator of the Wnt/β-catenin signaling pathway in OV

**Fig. 4:**
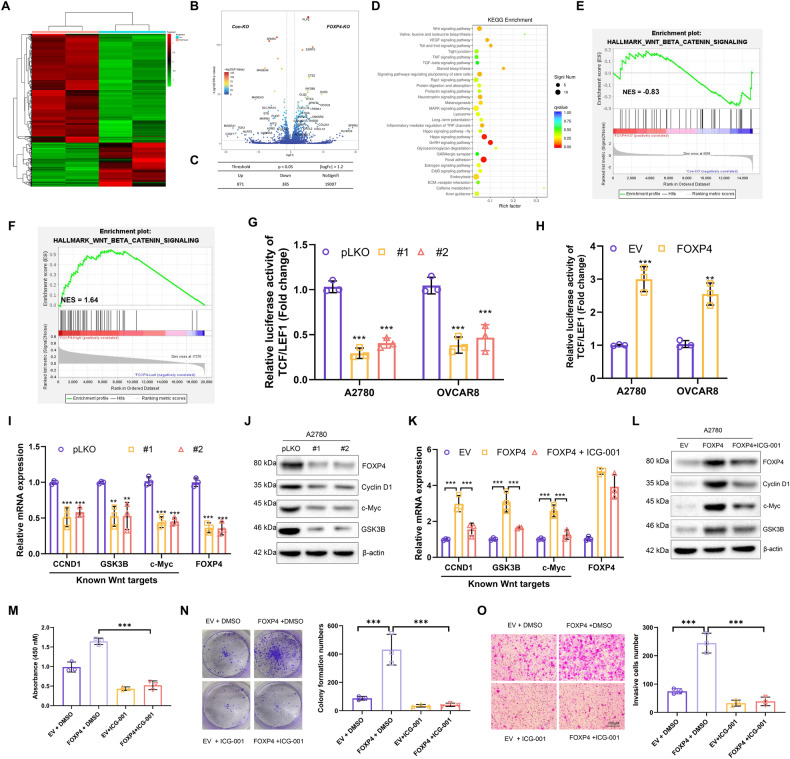
FOXP4 functions as a positive regulator of the Wnt/β-catenin signaling pathway in OV. **A** The RNA-seq data of 426 OV samples from TCGA was subjected to GSEA, the HALLMARK_WNT_BETA_CATENIN_SIGNALING was enriched in high FOXP4 expression group. **B** Differentially expressed genes in WT and FOXP4-KO cell lines were analyzed, and volcano plots are expressed as up-regulated and down-regulated genes with |LogFC| >1.2 and *P* < 0.05. **C** The table indicates that 871 genes are up-regulated and 385 genes are down-regulated. **D** KEGG analysis of the cells in FOXP4-KO revealed significant changes in the Wnt pathway. **E** GSEA plot of enriched pathways in RNA-seq data from FOXP4-KO and WT. **F** GSEA plot of enriched pathways in RNA-seq data of 426 OV samples from TCGA. **G** The luciferase activities of TCF/LEF1 in A2780 and OVCAR8 cells transfected with pLKO or FOXP4-shRNAs were measured, *n* = 3. ***p < 0.001 vs pLKO. **H** The luciferase activities of TCF/LEF1 in A2780 and OVCAR8 cells transfected with EV or FOXP4 were measured, *n* = 3. **p < 0.01 vs pLKO, ***p < 0.001 vs pLKO. **I** Real-time PCR was used to measure mRNA levels of FOXP4, CCND1, GSK3B, and c-Myc in A2780 cells stably expressing either control vector (pLKO) or shRNAs targeting FOXP4, *n* = 3. **p < 0.01 vs pLKO, ***p < 0.001 vs pLKO. **J** Immunoblot analysis was performed to evaluate the protein levels of FOXP4, CCND1, GSK3B, and c-Myc in cells stably expressing pLKO or shRNAs targeting FOXP4. **K** Real-time PCR was used to determine the expression of FOXP4 and Wnt/β-catenin target genes in A2780 cells transfected with EV or FOXP4 with/without ICG-001 treatment for 24 h, *n* = 3. ***p < 0.001 vs pLKO. **L** Immunoblot analysis was used to detect the protein levels of FOXP4, CCND1, GSK3B, and c-Myc in A2780 cells with stable expression of EV and FOXP4 or overexpression of FOXP4 or ICG-001. **M** Cell viability determination was performed on the cells stably expressing EV and FOXP4 after treatment with DMSO or ICG-001, *n* = 3. ***p < 0.001. **N** The Colony formation numbers of cells in (M) in colony experiments, *n* = 3. ***p < 0.001. **O** Relative invasion rates of cells in (M) in a transwell assay, *n* = 3. ***p < 0.001.

We initiated our investigation into the downstream signaling pathways regulated by FOXP4 by creating FOXP4 knockout (KO) cell lines from 293T cells, utilizing CRISPR-Cas9 technology. Following this, high-throughput RNA sequencing was conducted on samples from FOXP4 wild-type (WT) and FOXP4 KO cells. A heatmap illustrated significant gene expression between the FOXP4 KO group and the control group (Fig. 4A). Genes were classified as differentially expressed (DEGs) based on criteria of |LogFC| > 1.2 and *p* < 0.05. Compared to FOXP4 WT cells, FOXP4 KO cells showed upregulation of 871 genes and downregulation of 385 genes (Fig. 4B, C). Analysis of the downregulated gene set with the Kyoto Encyclopedia of Genes and Genomes (KEGG) revealed significant alterations in several signaling pathways, with the most significant being the Wnt/β-catenin signaling pathway (Fig. 4D). Next, the differential gene sets of the two FOXP4 groups were enriched and analyzed, and the results showed that the Wnt/β-catenin signaling pathway was negatively correlated with the FOXP4 KO group (Fig. 4E). Moreover, ovarian cancer samples from the TCGA database were categorized into high and low FOXP4 expression groups for GSEA analysis. The results showed that Wnt/β-catenin signaling pathway was enriched in the FOXP4 high expression group (Fig. 4F). Considering the Wnt/β-catenin signaling pathway’s pivotal role in tumor cell function, we delved deeper into FOXP4’s potential to influence this pathway’s activity. To do so, we evaluated changes in luciferase activity of TCF/LEF1 in A2780 and OVCAR8 cells with or without elimination of FOXP4, respectively. TCF/LEF1 is a recognized downstream target gene of the Wnt/β-catenin signaling pathway. We found that silencing FOXP4 significantly decreased TCF/LEF1 luciferase activity (Fig. 4G). Conversely, overexpression of FOXP4 significantly increased TCF/LEF1 luciferase activity in both OV cell lines (Fig. 4H). Importantly, silencing FOXP4 led to reduced transcription of several established Wnt/β-catenin target genes, including CCND1, GSK3B, and c-Myc (Fig. 4I). The corresponding protein expression was also decreased (Fig. 4J). Conversely, the expression of these genes was also increased in FOXP4-overexpressing cells, which could be inhibited by the Wnt pathway inhibitor ICG-001 (Fig. 4K, L). Furthermore, ICG-001 was able to inhibit FOXP4 overexpression-induced cell proliferation (Fig. 4M), colony formation (Fig. 4N), and invasion (Fig. 4O) in A2780 cells. The findings indicate FOXP4’s ability to activate the Wnt/β-catenin signaling pathway in ovarian cancer, which is vital for FOXP4’s oncogenic role.

### PTK7 is a transcriptional target of FOXP4 in OV

**Fig. 5:**
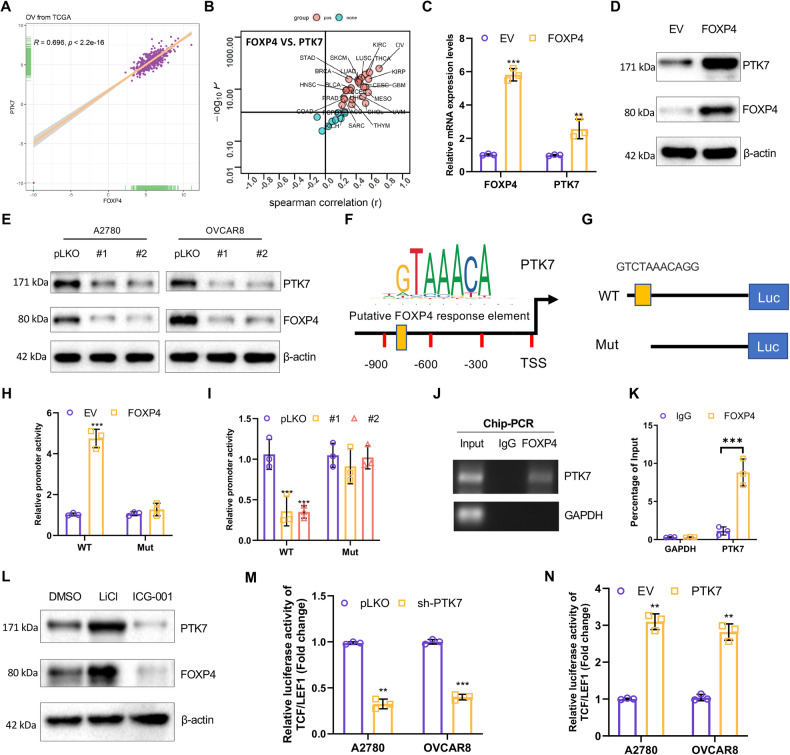
PTK7 is a transcriptional target of FOXP4 in OV. **A** The mRNA correlation between FOXP4 and PTK7 in OV from TCGA database. **B** The spearman correlation between the expression of FOXP4 and PTK7 in all tumor types in TCGA. Red represents positive expression and green represents negative expression. **C** Real-time PCR analysis of FOXP4 and PTK7 mRNA levels in A2780 cells transfected with EV or FOXP4 expression vector, *n* = 3. **p < 0.01 vs EV, ***p < 0.001 vs EV. **D** Immunoblot analysis showing FOXP4 and PTK7 protein expression in A2780 cells transfected with EV or FOXP4. **E** Immunoblot analysis of FOXP4 in A2780 and OVCAR8 cells stably expressing either pLKO or shRNAs targeting FOXP4. **F** Schematic diam shows human PTK7 gene promoter and a putative FOXP4 binding site. TSS: transcription start site. **G** Schematic diam shows the human PTK7 WT and Mut promoters. The potential binding sites for FOXP4 were highlighted with yellow. **H** The activities of the human PTK7 WT and Mut promoters in 293T cells transfected with EV or FOXP4 was measured, *n* = 3. ***p < 0.001 vs EV. **I** Two shFOXP4s were co-transfected with the indicated plasmid into 293T cells for 36 h. The luciferase activity was then measured, *n* = 3. ***p < 0.001 vs pLKO. **J**, **K** Chip-PCR assay detecting FOXP4 binding to the human PTK7 promoter in A2780 cells expressing stable FOXP4, *n* = 3. ***p < 0.001 vs IgG. **L** A2780 cells were treated with ICG-001 or LiCl to detect PTK7 protein levels. **M** The luciferase activities of TCF/LEF1 in A2780 and OVCAR8 cells transfected with pLKO or shRNAs-PTK7 were measured, *n* = 3. **p < 0.01 vs pLKO, ***p < 0.001 vs pLKO. **N** The luciferase activities of TCF/LEF1 in A2780 and OVCAR8 cells transfected with EV or PTK7 were measured, *n* = 3. **p < 0.01 vs EV.

To unravel the molecular mechanism through which FOXP4 enhances the activity of the Wnt/β-catenin signaling pathway, we emined all genes co-expressed with FOXP4 from the TCGA OV database. Of the genes whose function is related to the Wnt/β-catenin signaling pathway, we focused on PTK7, as it showed the most significant correlation with FOXP4 in OV and most other cancer types in TCGA (Fig. 5A, B). To test if PTK7 is regulated by FOXP4, we engineered OV cells to overexpress FOXP4. We found that FOXP4 overexpression significantly induced both transcription and protein expression of PTK7 (Fig. 5C, D). Conversely, silencing FOXP4 with two different shRNAs resulted in a consistent reduction in PTK7 protein expression (Fig. 5E). Additionally, utilizing the JASPAR online database, our finding of a response element for FOXP4 in the promoter region of PTK7 would suggest to us that FOXP4 acts as a transcription factor that may induce PTK7 transcription (Fig. 5F). To explore this further, we constructed two luciferase reporter vectors: one harboring the putative FOXP4 response element and another without it (Fig. 5G). Consistent with our hypothesis, FOXP4 overexpression triggered a 4-5 fold increase in the activity of the reporter containing the FOXP4 response element (Fig. 5H). Conversely, silencing FOXP4 expression led to a significant loss of PTK7 promoter activity, and the deletion of the FOXP4 response element abolished the response of the PTK7 promoter to FOXP4 expression (Fig. 5I). Furthermore, Chip assays confirmed FOXP4’s binding to the PTK7 promoter (Fig. 5J, K, Supplementary Fig. 4A, B). Subsequently, we examined the expression level of PTK7 by manipulating β-catenin signaling activity. The results showed that the expression of PTK7 increased with the activation of β-catenin pathway (Fig. 5L). Meanwhile, overexpression of PTK7 induced an increase in TCF/LEF1 luciferase activity (Fig. 5N). In contrast, knockdown of PTK7 significantly reduced TCF/LEF1 luciferase activity (Fig. 5M). Therefore, these findings elucidate that FOXP4 directly regulates PTK7 expression at the transcriptional level, reinforcing FOXP4’s role in modulating the Wnt/β-catenin signaling pathway.

### FOXP4’s oncogenic potential in OV cells requires PTK7

**Fig. 6:**
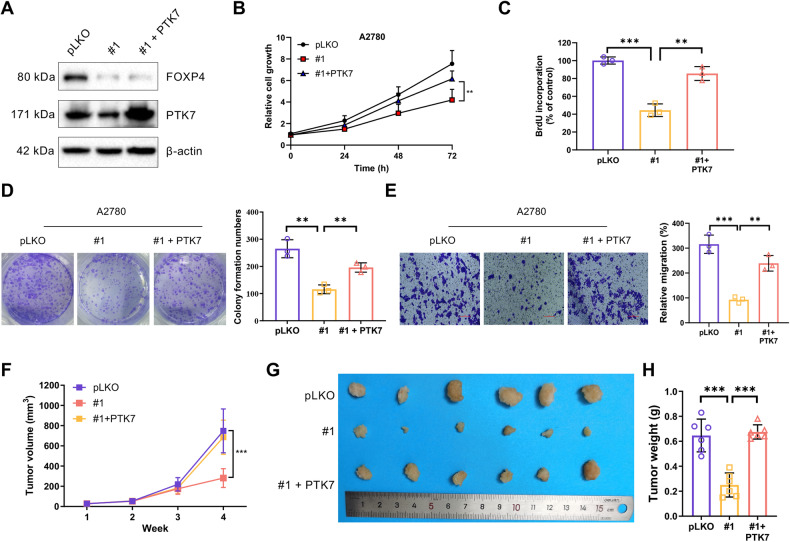
FOXP4’s oncogenic potential in OV cells requires PTK7. **A** PTK7 was retrovirally infected into A2780 cells stably expressing FOXP4-shRNA. Lysates of A2780 cells with pLKO, sh-FOXP4, and sh-FOXP4 + PTK7 were immunoblotted with the indicated antibodies. **B** The cell growth curve of cells from (**A**), *n* = 3. **p < 0.01. **C** Incorporation of BrdU in cells from (**A**) was measured by ELISA, *n* = 3. **p < 0.01, ***p < 0.001. **D** Clonogenic assay of cells in (**A**), *n* = 3. *p < 0.05, **p < 0.01, ***p < 0.001. **E** Relative migration rates of cells in (**A**) in a transwell assay, *n* = 3. **p < 0.01, ***p < 0.001. **F** A2780 cells stably expressing pLKO or sh-FOXP4 and stably expressing sh-FOXP4 + PTK7 were inoculated subcutaneously in nude mice, respectively, and the folded line graphs indicate the average volume of tumors over time, *n* = 6. ***p < 0.001. **G** Image representing the tumor on day 28, *n* = 6. H Bar graphs represent the weight of the tumor on day 28, *n* = 6. ***p < 0.001.

To investigate whether PTK7 mediates the tumor-promoting function of FOXP4 in OV, we generated FOXP4 knockout cells with stable expression of exogenous PTK7 (Fig. 6A). The expression level of exogenous PTK7 matched that of the control group, enabling a direct comparison of their biological functions. The effect of FOXP4 knockdown on the reduction of OV cell proliferation and colony formation was shown to be significantly decreased by increased PTK7 expression (Fig. 6B–D). Additionally, PTK7 overexpression was observed to augment cell migration in FOXP4-silenced cells (Fig. 6E). Given that the invasive ability of cancer cells is closely linked to the process of epithelial-mesenchymal transition (EMT), we examined the expression of EMT markers in detail. The results showed that knockdown of CTNNB1 not only reduced the downstream expression levels of FOXP4 and PTK7, but also significantly increased the expression of E-cadherin, and significantly decreased the expression of N-cadherin and vimentin. This series of changes revealed that β-catenin/FOXP4/PTK7 signaling pathway may affect the invasion ability of ovarian cancer cells by regulating EMT process (Supplementary Fig. 5A). In mouse xenograft tumor models, FOXP4 knockdown diminished tumor growth, yet reintroducing PTK7 partially reversed this effect (Fig. 6F–H). These findings were further supported by Western blot analysis, which showed decreased PTK7 protein levels in the mouse tumor samples following FOXP4 knock down (Supplementary Fig. 5B).

**Fig. 7:**
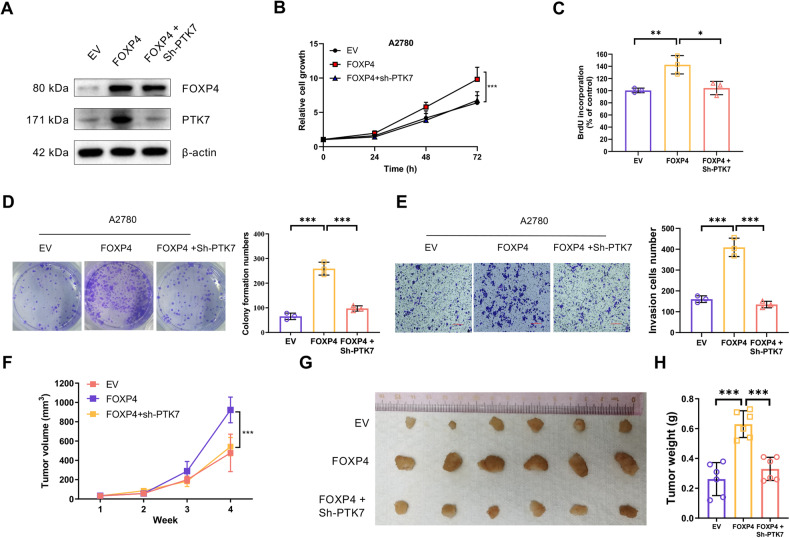
FOXP4’s oncogenic potential in OV cells requires PTK7. **A** The FOXP4 retrovirus was infected into A2780 cells stably expressing sh-PTK7, and A2780 cell lysates of pLKO, FOXP4, and FOXP4+sh-PTK7 were immunobloted with the indicated antibodies. **B** The cell growth curve of cells from (**A**), *n* = 3. ***p < 0.001. **C** Incorporation of BrdU in cells from (**A**) was measured by ELISA, *n* = 3. *p < 0.05, **p < 0.01. **D** Clonogenic assay of cells in (**A**), *n* = 3. ***p < 0.001. **E** Relative invasion rates of cells in (**A**) in a transwell assay, *n* = 3. ***p < 0.001. **F** A2780 cells stably expressing EV or FOXP4 or FOXP4 infected with sh-PTK7 in FOXP4 were inoculated subcutaneously in nude mice, respectively, and the folded line graphs indicate the average volume of tumors over time, *n* = 6. ***p < 0.001. **G** Image representing the tumor on day 28, *n* = 6. **H** Bar graphs represent the weight of the tumor on day 28, *n* = 6. ***p < 0.001.

**Fig. 8:**
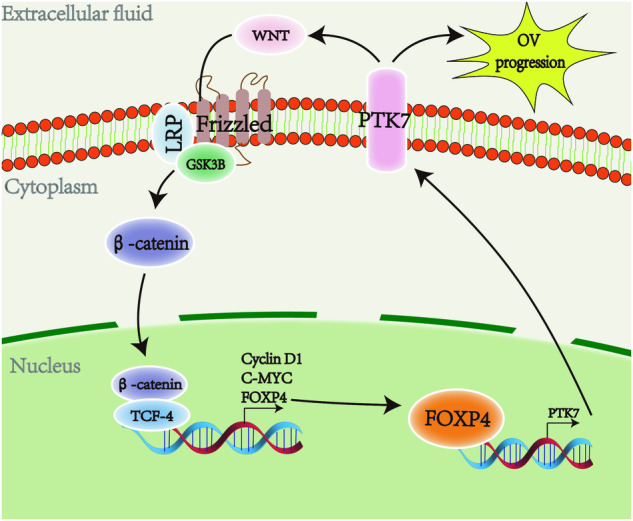
Working model. In this working model, we depict a signaling pathway involving Wnt/β-catenin/TCF4-FOXP4-PTK7. FOXP4 acts as a transcriptional target of Wnt/β-catenin/TCF4 signaling and enhances the transcription of PTK7, consequently promoting the development and progression of OV tumors.

Subsequently, we created OV cells with stable overexpression of FOXP4 and concurrent silencing of PTK7 (Fig. 7A). As expected, FOXP4 overexpression significantly enhanced cell viability, invasion, and migration. Silencing PTK7 considerably obstructed these malignant behaviors (Fig. 7B–F). This observation was further corroborated in a xenograft tumor experiment with mice, where tumor growth driven by FOXP4 overexpression was markedly reduced following PTK7 silencing (Fig. 7G–I). Finally, we assessed the prognostic relationship between β-catenin/FOXP4/PTK7 signature and OV patients and found that patients with high expression of β-catenin/FOXP4/PTK7 signature were associated with poorer Overall survival (OS), Progression-free survival (PFS) and Post-progression survival (PPS) (Supplementary Fig. 6A–C). Taken together, these results suggest that PTK7 plays a critical role in facilitating FOXP4’s tumor-promoting effects in OV cells, highlighting the importance of PTK7 suppression in this context (Fig. 8).

## Discussion

Detecting ovarian cancer early poses significant challenges, often resulting in diagnoses at advanced stages where therapeutic interventions frequently fall short of achieving complete remission. The quest for effective treatments for ovarian cancer is intricately intertwined with unraveling its molecular mechanisms. In this investigation, we delved into the intricate pathogenesis of ovarian cancer, shedding light on the pivotal role of FOXP4 as a potent oncogenic driver. Our findings reveal aberrant upregulation of FOXP4 mRNA, correlating with poor prognostic outcomes in ovarian cancer tissues, through integration of data from the TCGA and GEO databases. Activation of the Wnt signaling pathway is concomitant with escalated FOXP4 protein expression, a phenomenon further substantiated by our verification of Wnt downstream transcription factor TCF4’s role in inducing this upregulation. FOXP4 emerges as a novel transcriptional target of Wnt/β-catenin/TCF4 signaling. Suppression of FOXP4 markedly attenuates tumor cell proliferation and inhibits tumor growth, with implications potentially linked to the EMT pathway. Furthermore, FOXP4 drives the mRNA expression of proteins pertinent to the Wnt/β-catenin signaling pathway, a pathway widely acknowledged for its pivotal regulatory role in tumor cell proliferation, migration, and invasion. Experimental evidence underscores FOXP4’s role as a transcriptional activator of PTK7, thereby facilitating ovarian cancer progression. Significantly, silencing PTK7 mitigates these malignant traits. In essence, FOXP4 emerges as a pivotal transcriptional target within the Wnt/β-catenin/TCF4 signaling axis, regulating the downstream transcription factor PTK7 and thereby fostering tumor proliferation, migration, and invasiveness.

The Wnt signaling pathway plays a pivotal role in a myriad of developmental and pathological phenomena, including physical maturation [23], cancer [24], and degenerative conditions [25]. Beyond these established functions, its involvement in novel physiological pathways, such as blood pressure regulation, underscores its potential to redefine hypertension management strategies [26]. Recent studies underscore the critical role of aberrant activity or mutations within the genes constituting the Wnt signaling network as key influencers in oncogenesis [27], encompassing colorectal [28] and a broad spectrum of other cancer types. Evidence firmly positions Wnt signaling as a fundamental arbiter of tissue homeostasis and a carcinogenic force across various malignancies [29]. Disruptions within the canonical Wnt/β-catenin axis are linked to a diverse array of cancers, where inhibiting this pathway has yielded promising anti-tumor outcomes [30]. Furthermore, research highlighting the interaction between PTK7/Otk and Wnt components, leading to the inhibition of standard Wnt signaling pathways [19], enriches our understanding. In the contemporary arena of targeted therapy, the Wnt signaling axis has risen as a pivotal target for anti-cancer drug development efforts. Yet, a comprehensive grasp of its molecular intricacies remains out of reach. Deciphering these intricate mechanisms promises to significantly propel forward our comprehension and the command over the Wnt pathway as a cancer therapeutic target.

PTK7, a multifaceted transmembrane receptor, plays a pivotal role in a vast array of developmental dynamics and the preservation of tissue equilibrium. Recent research underscores its critical biological functions across diverse cancer types. Notably, PTK7 has been identified as a prognostic marker for colon cancer, suggesting its utility in forecasting the disease onset [31]. Additionally, PTK7 serves as a cellular receptor intricately involved with the Wnt signaling pathway, influencing its activity. However, the interplay between PTK7 and Wnt signaling diverges across different cancer and disease contexts. In the case of epithelial ovarian cancer, a lack of PTK7 expression correlates with advanced disease stages and the presence of metastases [32]. Regulatory mechanisms involving the transcription factor FOXP4 have been shown to modulate PTK7, contributing to the malignant progression of ovarian cancer, though the specifics of downstream signaling regulation remain uncertain.

Thus, several critical inquiries arise: Is there a direct linkage between PTK7 and the onset of ovarian cancer? Do the downstream effects of PTK7 implicate Wnt signaling proteins or other regulatory elements? These questions underscore gaps in our understanding, necessitating further experimental validation. With ongoing research, PTK7 holds promise as a potential therapeutic target not only for ovarian cancer but potentially for a broader range of tumor types.

In conclusion, this investigation pioneers the recognition of FOXP4’s substantial upregulation in ovarian cancer and its direct correlation with adverse outcomes, underscoring the imperative for comprehensive insight into the molecular intricacies of ovarian cancer. We have pinpointed FOXP4 as an innovative target gene within the Wnt/β-catenin/TCF4 signaling cascade. From a mechanistic standpoint, the activation of the Wnt/β-catenin pathway augments FOXP4 transcription, thereby facilitating the proliferation and migration of ovarian cancer cells. Furthermore, the escalated expression of FOXP4 significantly enhances PTK7 levels. This, in conjunction with PTK7’s activation of the β-catenin pathway, establishes a β-catenin/FOXP4/PTK7 positive feedback loop, culminating in the accelerated advancement of ovarian cancer. These groundbreaking discoveries furnish new vistas into the molecular underpinnings of ovarian cancer and highlight the possibility that interventions targeting this pathway may herald novel therapeutic avenues for patients with ovarian cancer.

### Supplementary information

Supplementary Figure

supplementary legends

Full and uncropped western blot

## Data Availability

The experimental data sets generated and/or analyzed during the current study are available from the corresponding author upon reasonable request. No applicable resources were generated during the current study.
