# Supplementary material for: FOXP4-mediated induction of PTK7 activates the Wnt/β-catenin pathway and promotes ovarian cancer development
Source: Cell Death Dis. 2024 May 13;15(5):332. doi: 10.1038/s41419-024-06713-7 (PMC11091054; doi:10.1038/s41419-024-06713-7)

Supplementary Figure 1

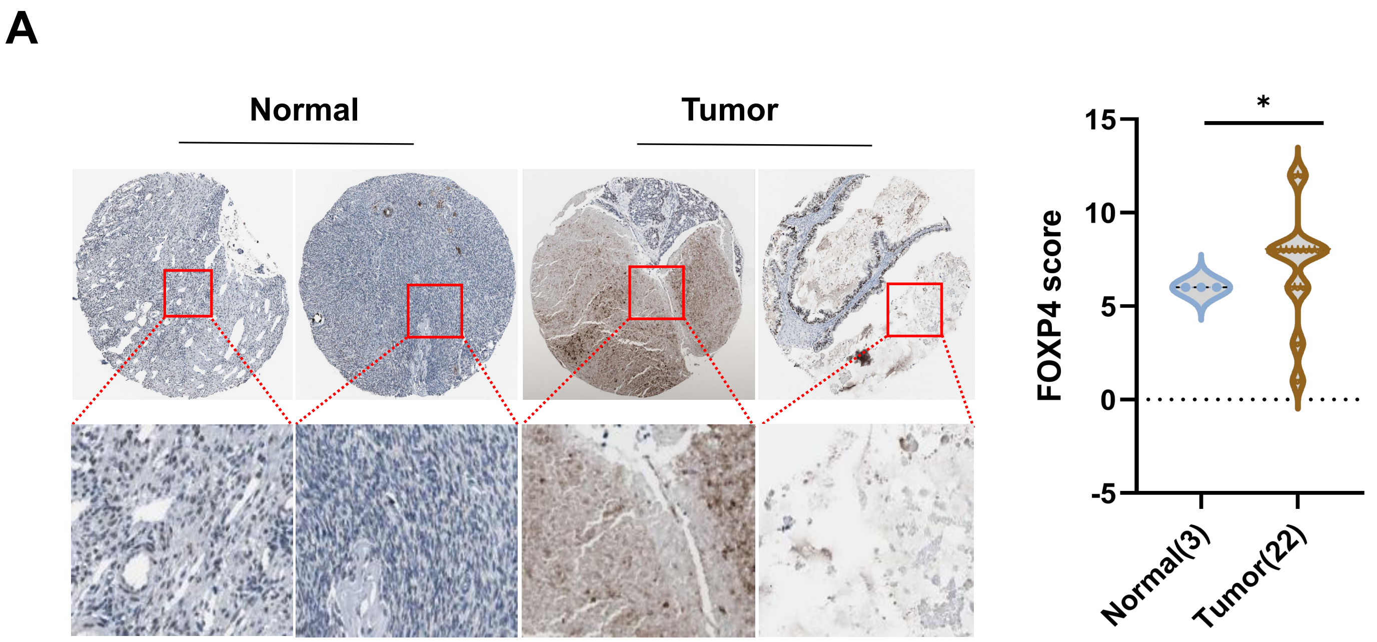

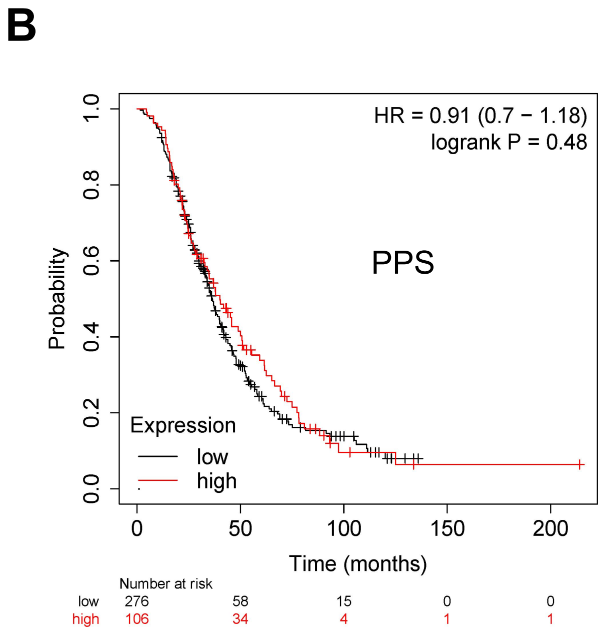

Supplementary Figure 2

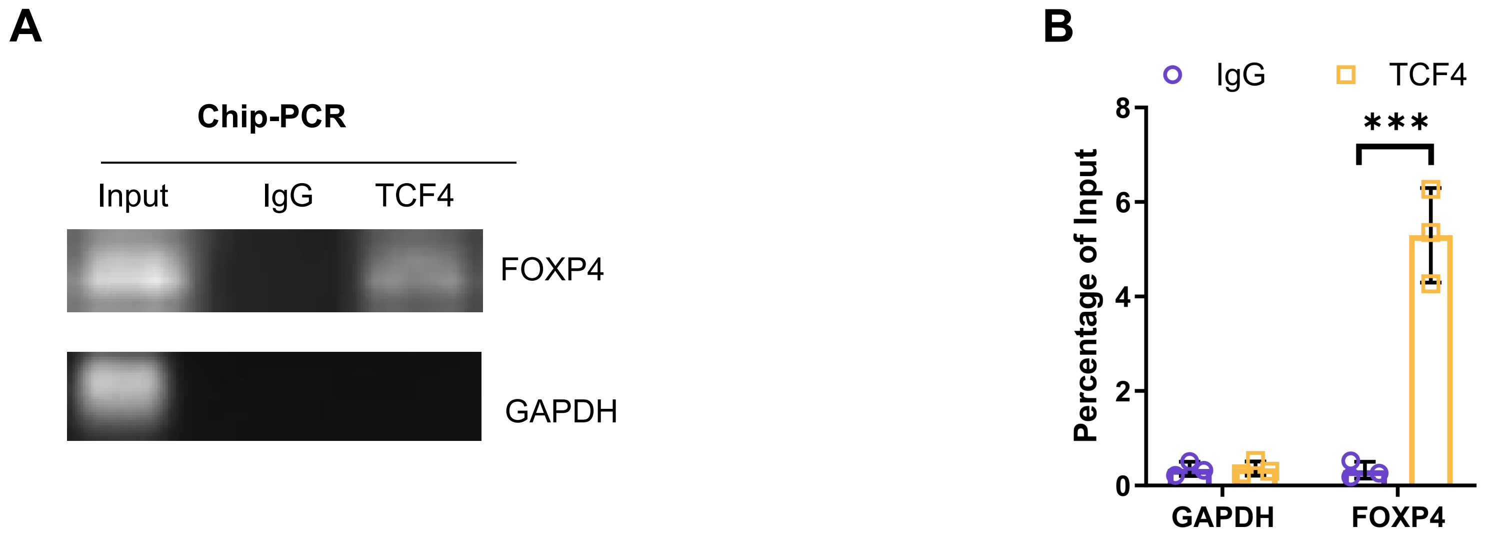

Supplementary Figure 3

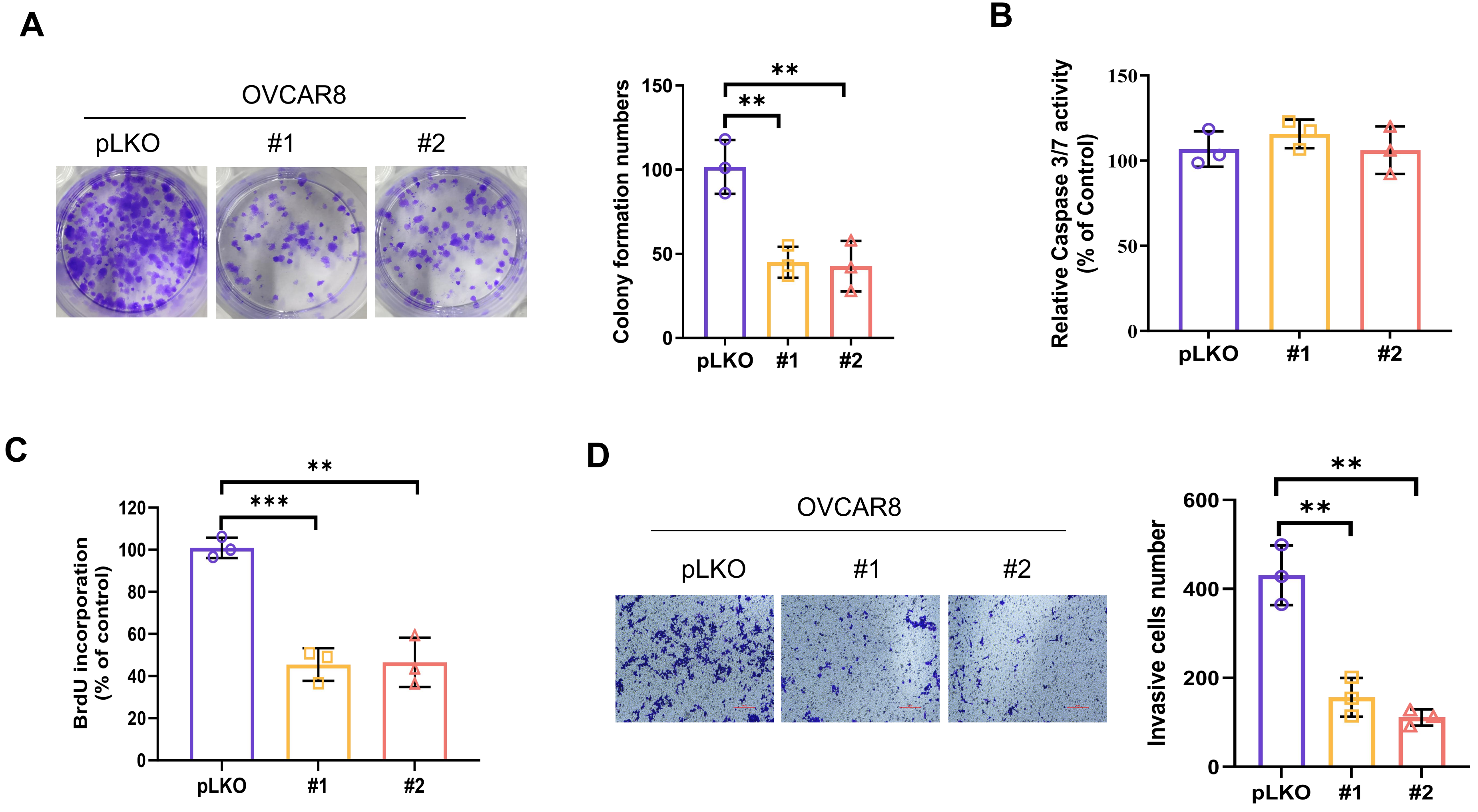

Supplementary Figure 4

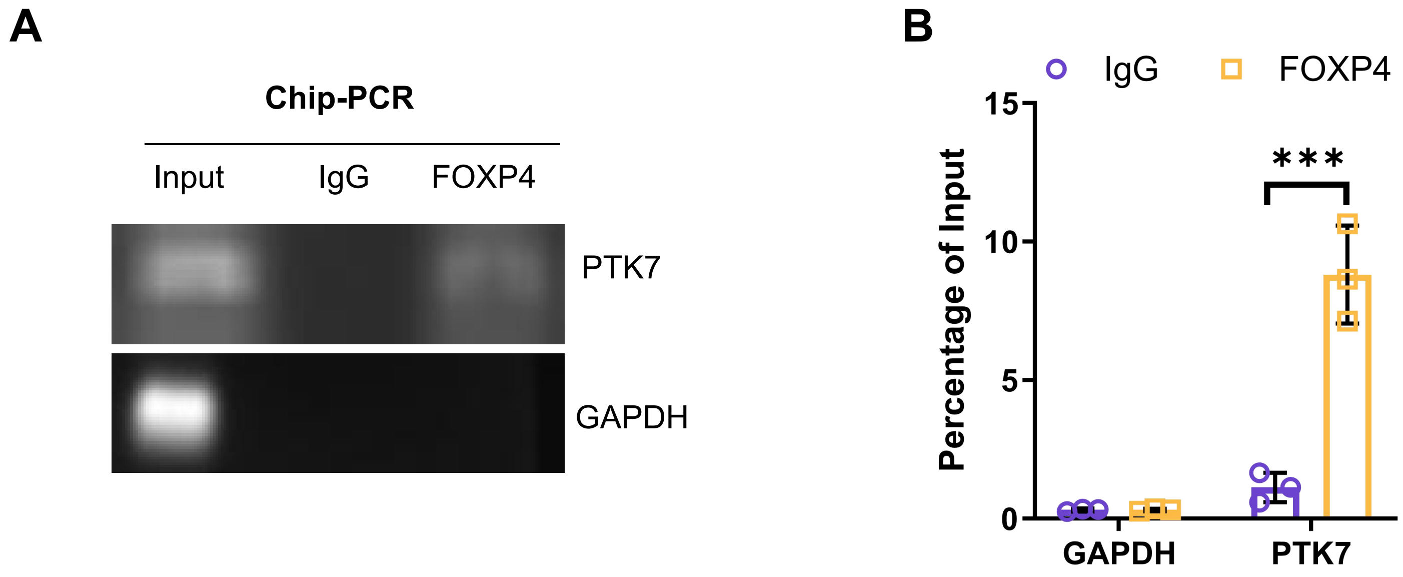

Supplementary Figure 5

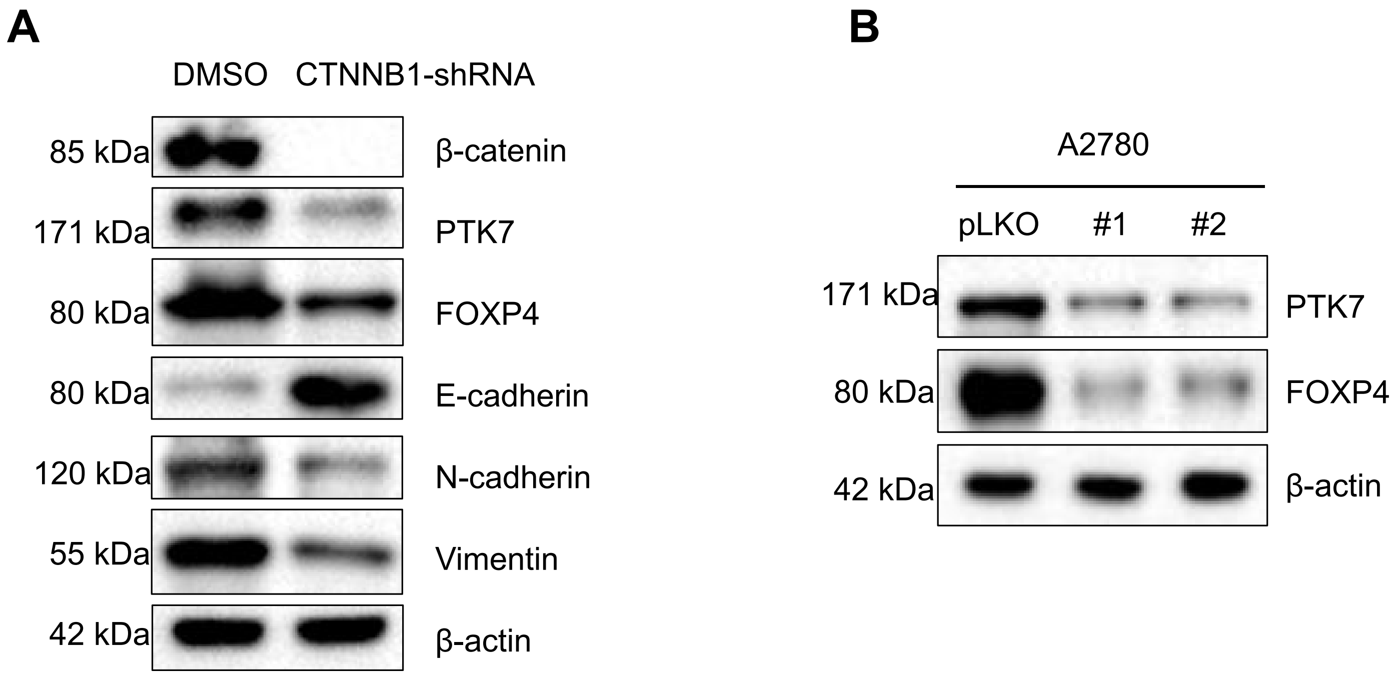

Supplementary Figure 6

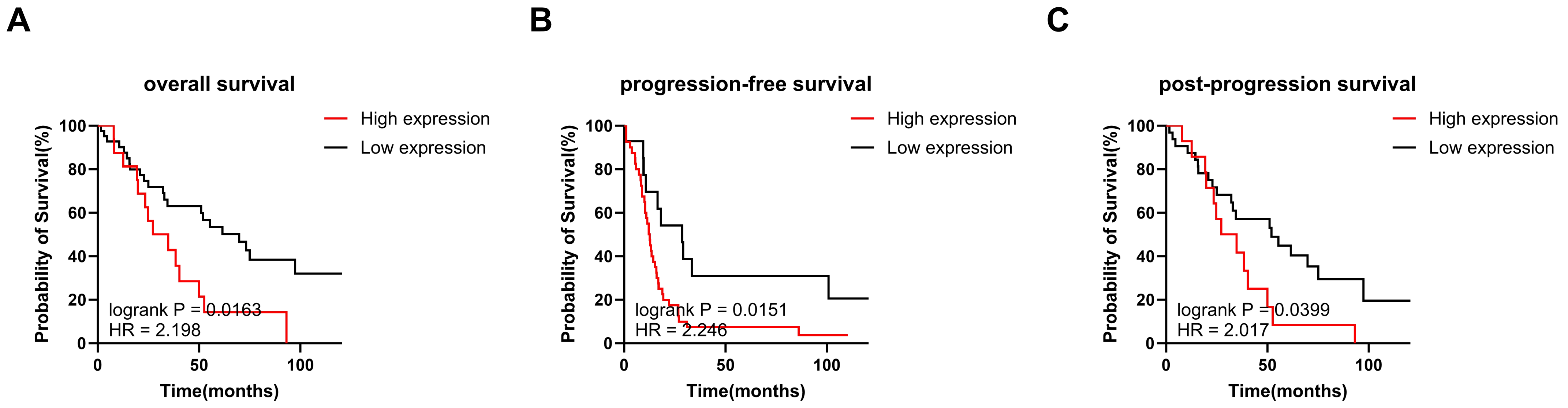

Supplement: Supplementary file 1 — Supplementary Figure [file 41419_2024_6713_MOESM1_ESM.pdf]
