# Supplementary material for: FOXP4-mediated induction of PTK7 activates the Wnt/β-catenin pathway and promotes ovarian cancer development
Source: Cell Death Dis. 2024 May 13;15(5):332. doi: 10.1038/s41419-024-06713-7 (PMC11091054; doi:10.1038/s41419-024-06713-7)
Supplement: Supplementary file 2 — supplementary legends [file 41419_2024_6713_MOESM2_ESM.docx]

**Supplementary Figure 1**

1. The representative IHC staining of FOXP4 protein in OV tissue (Ovarian serous cystadenocarcinoma) and normal tissue (ovary) from the Human Protein Atlas Project (<http://www.proteinatlas.org/>). **p* < 0.05.
2. The prognostic values of FOXP4 in OV patients. The survival curves comparing OV patients with high (red) and low (black) FOXP4 expression levels were plotted using the online Kaplan–Meier Plotter (<https://kmplot.com/>). PPS, post progression survival.

**Supplementary Figure 2**

A-B. OVCAR8 cells stably expressing TCF4 were subjected to Chip-PCR detection, the human GAPDH promoter was served as a negative control, n = 3. ****p* < 0.001 vs IgG.

**Supplementary Figure 3**

A. Clonogenic assay of OVCAR8 cells stably expressing pLKO or shRNAs against FOXP4, n = 3. ***p* < 0.01.

B. Caspase3 and Caspase7 activity was measured in OVCAR8 cells stably expressing pLKO or shRNAs against FOXP4. The y axis indicates the caspase3 and caspase7 activity over cell number. The value given for the caspase activity in control-infected cells was set as 100, n = 3.

C. Incorporation of BrdU in cells in (Figure 3B) was measured by ELISA, n = 3. ***p* < 0.01, ****p* < 0.001.

D. Relative invasion rates of cells in (Figure 3B) in a transwell assay, n = 3. ***p* < 0.01.

**Supplementary Figure 4**

A-B. OVCAR8 cells stably expressing FOXP4 were subjected to Chip-PCR detection, the human GAPDH promoter was served as a negative control, n = 3. ****p* < 0.001 vs IgG.

**Supplementary Figure 5**

A. The corresponding protein expression levels in A2780 cells transfected with Con-shRNA or shRNA-CTNNB1 were detected.

B. Immunoblot analysis of PTK7 protein levels in OV xenograft with pLKO or FOXP4-shRNAs.

**Supplementary Figure 6**

A-C. The GSE30161 dataset is based on the Overall survival (OS), Progression-free survival (PFS) and Post-progression survival (PPS) curves of ovarian cancer patients with β-catenin/FOXP4/PTK7 signature expression.
