# Supplementary material for: FOXP4-mediated induction of PTK7 activates the Wnt/β-catenin pathway and promotes ovarian cancer development
Source: Cell Death Dis. 2024 May 13;15(5):332. doi: 10.1038/s41419-024-06713-7 (PMC11091054; doi:10.1038/s41419-024-06713-7)

Figure2

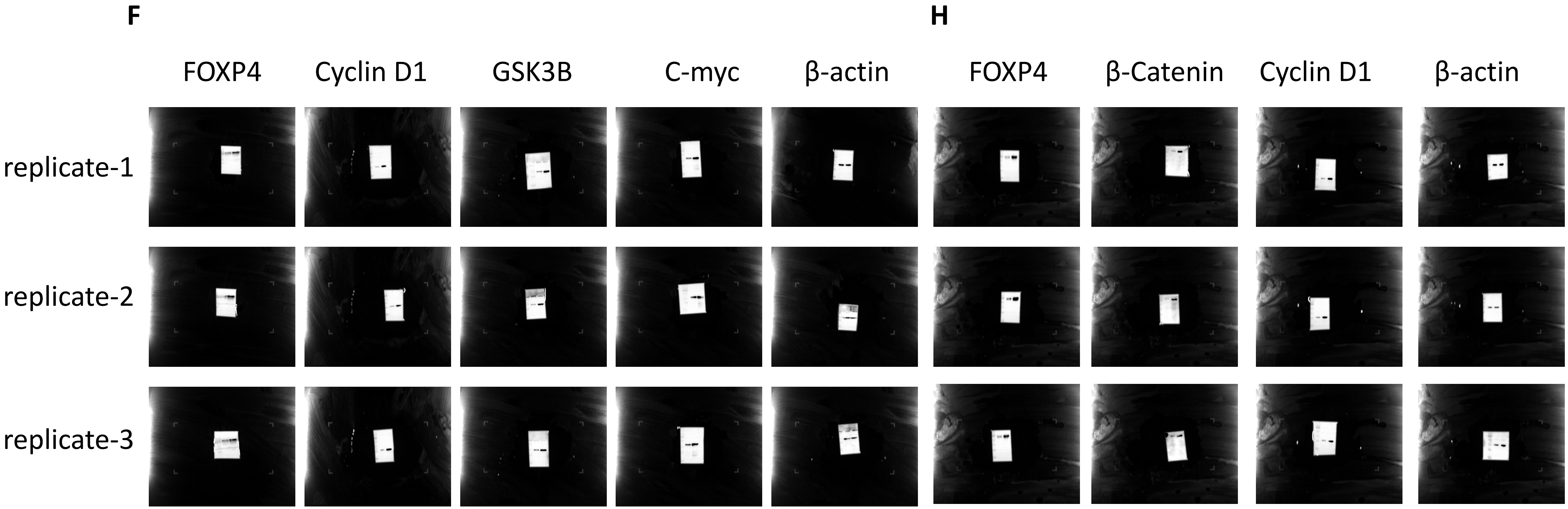
F
H
FOXP4
Cyclin D1
GSK3B
C-myc
β-actin
FOXP4
β-Catenin
Cyclin D1
β-actin
replicate-1
replicate-2
replicate-3

Figure2

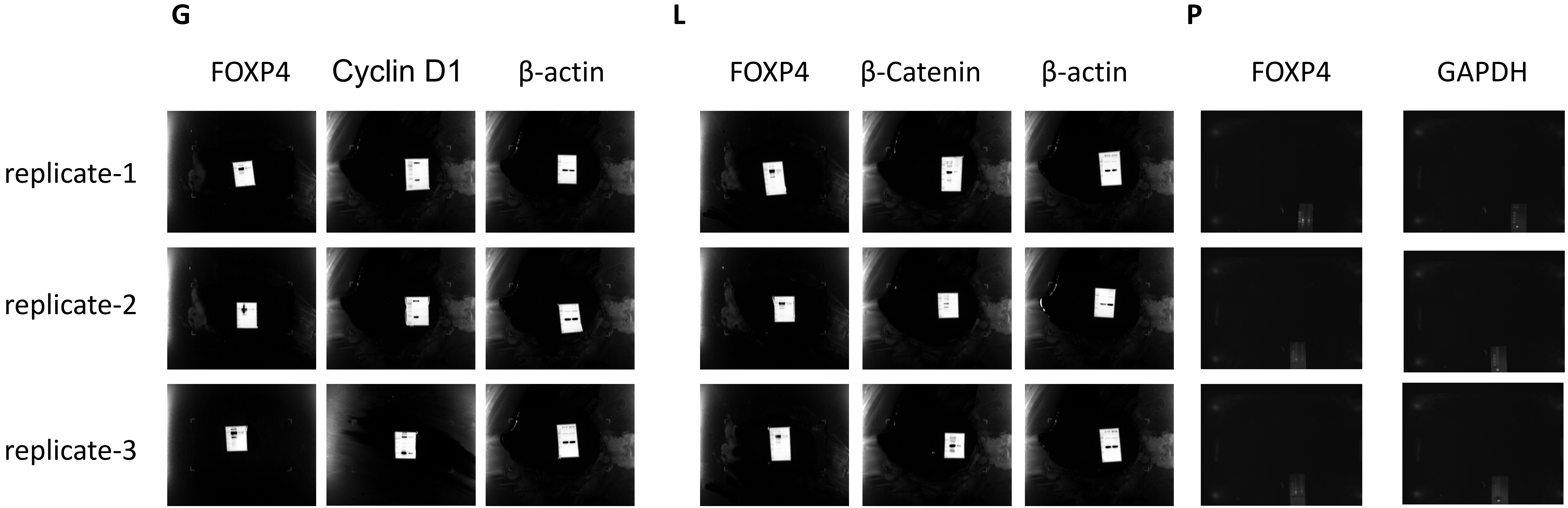
G
L
P
FOXP4
Cyclin D1
β-actin
FOXP4
β-Catenin
β-actin
FOXP4
GAPDH
replicate-1
replicate-2
replicate-3

Figure3

B

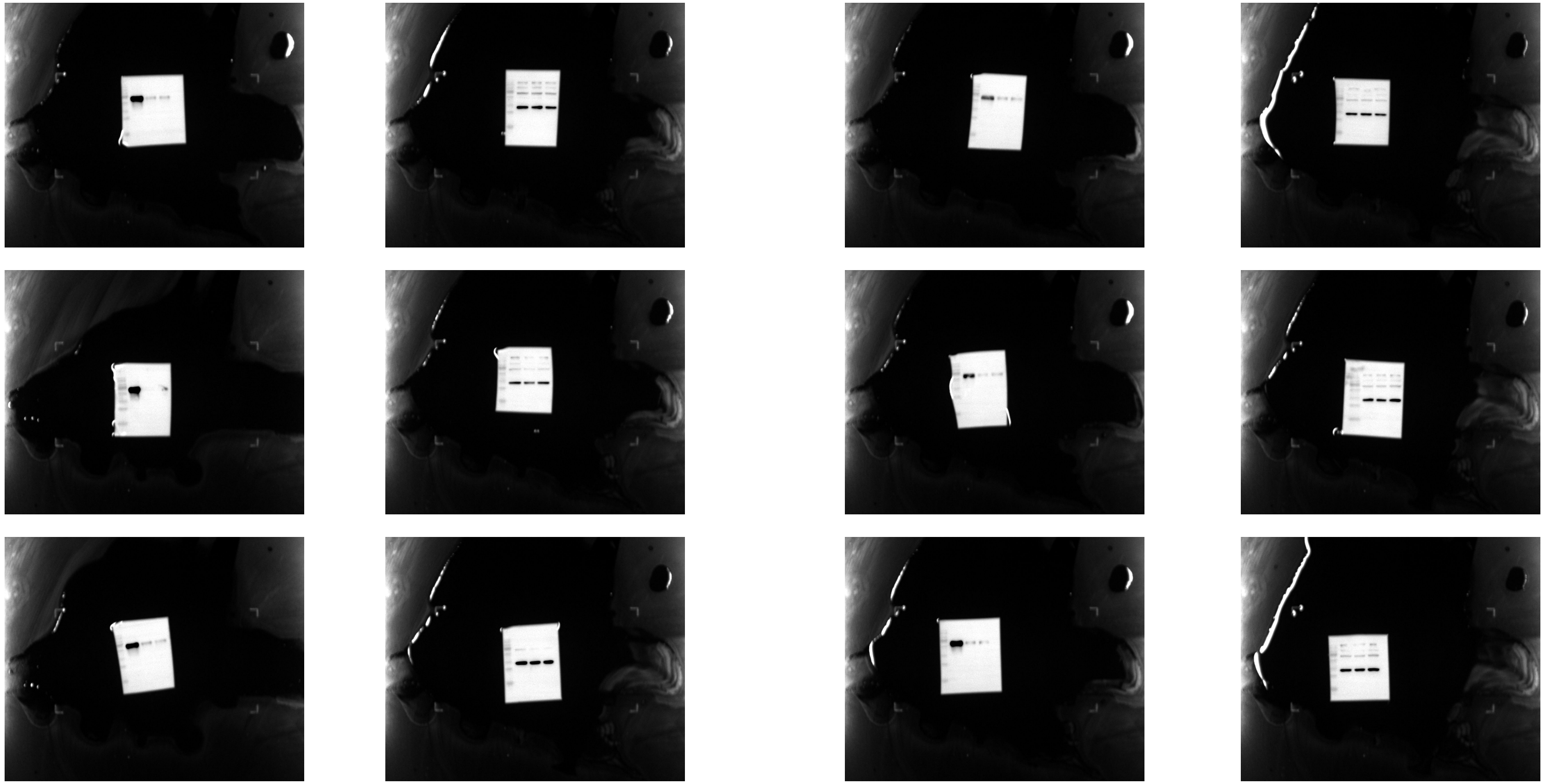
A2780
OVCAR8
FOXP4
β-actin
FOXP4
β-actin
replicate-1
replicate-2
replicate-3

Figure 4

J

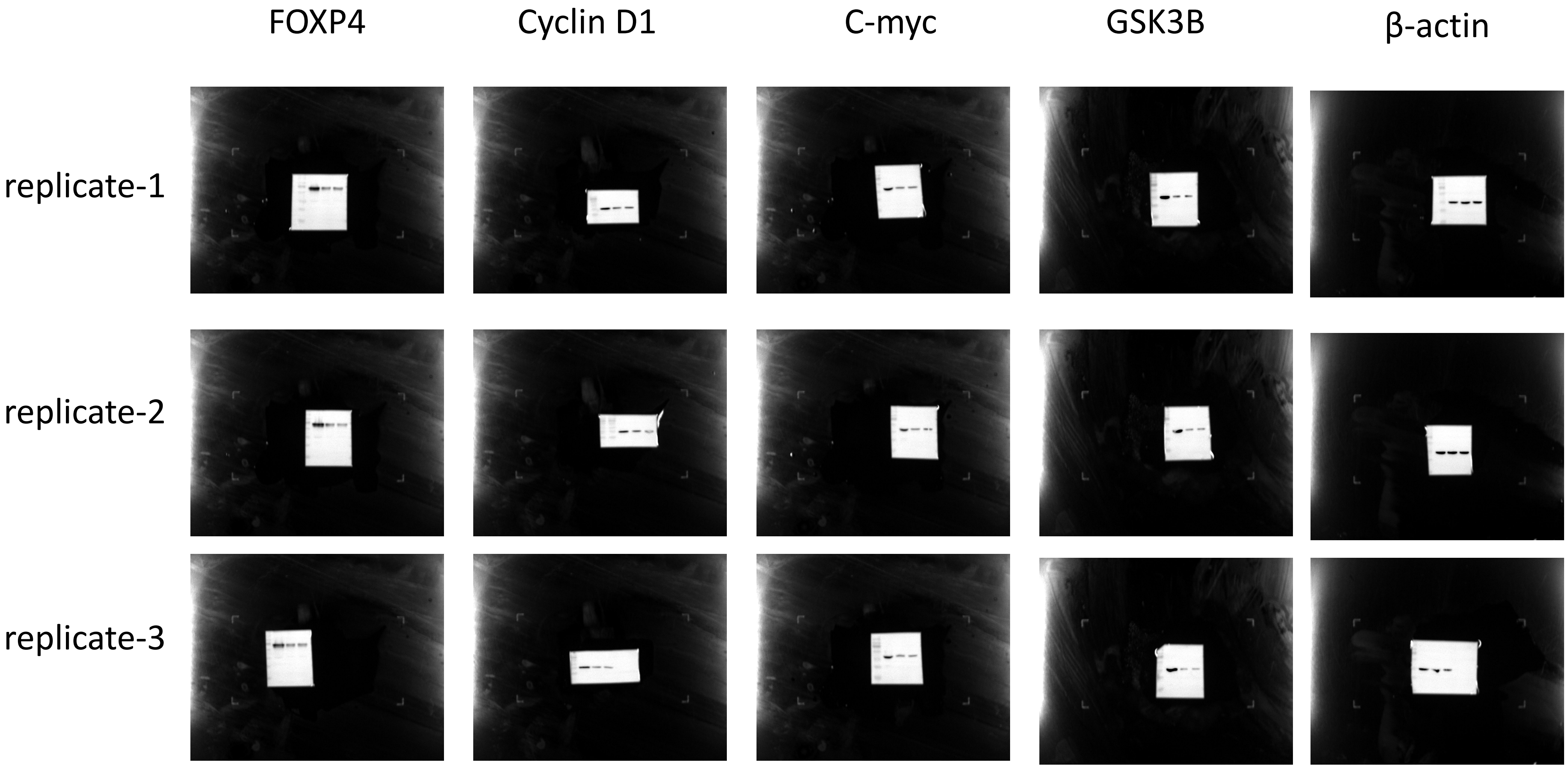
FOXP4
Cyclin D1
C-myc
GSK3B
β-actin
replicate-1
replicate-2
replicate-3

Figure 4

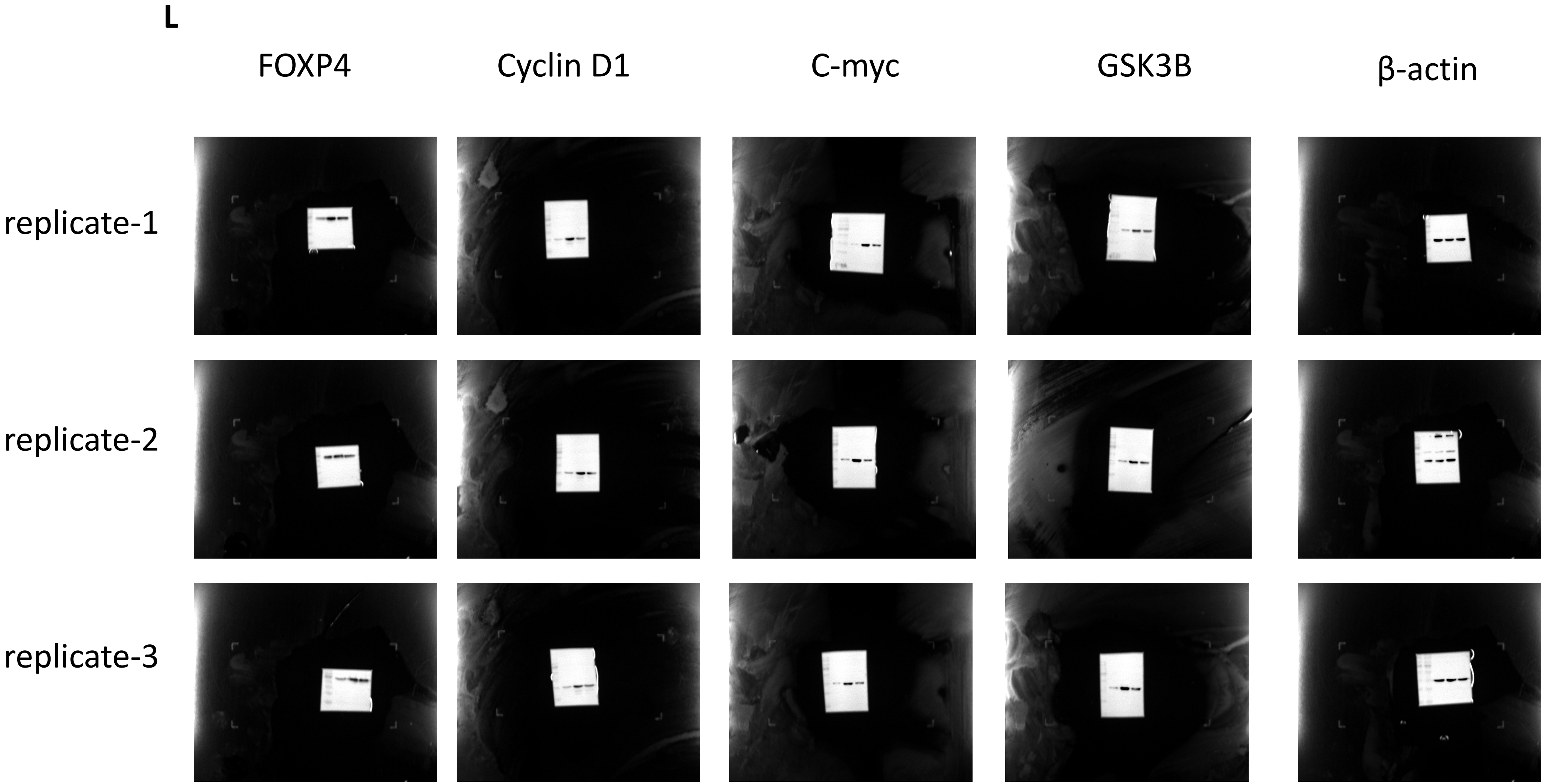
L
FOXP4
Cyclin D1
C-myc
GSK3B
β-actin
replicate-1
replicate-2
replicate-3

Figure5

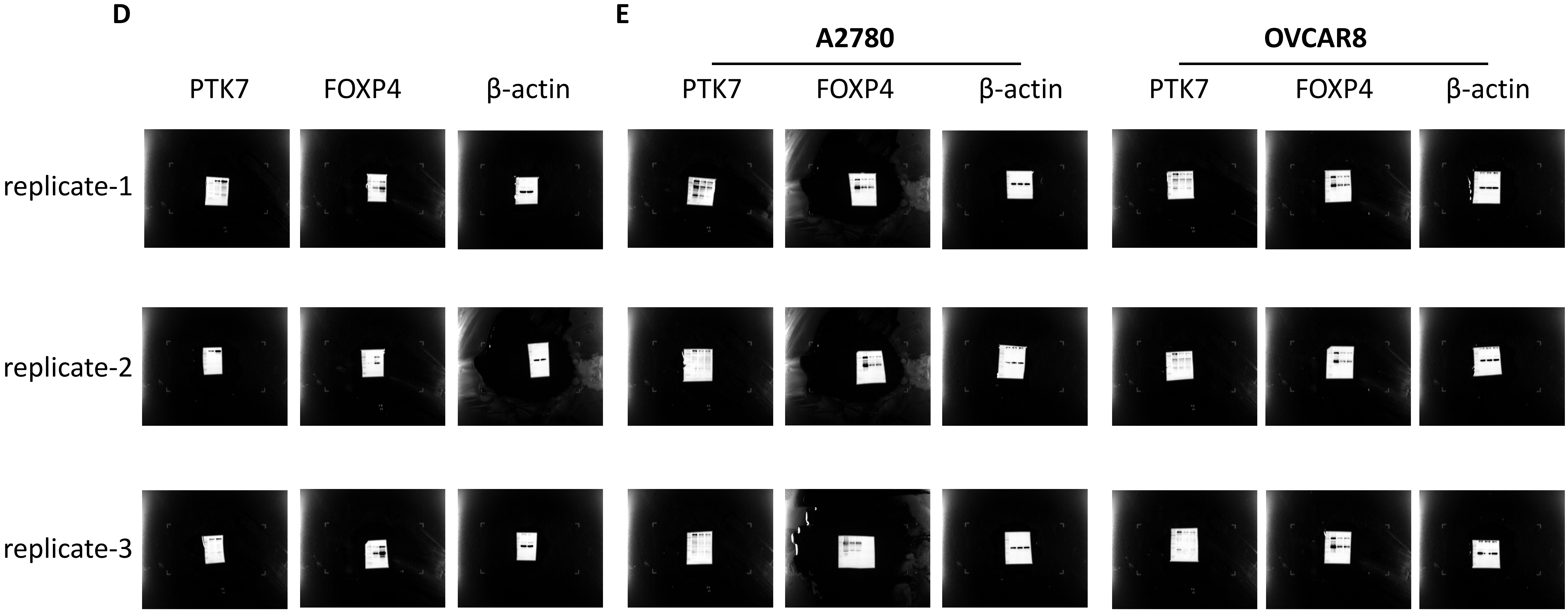
D
E
A2780
OVCAR8
PTK7
FOXP4
β-actin
PTK7
FOXP4
β-actin
PTK7
FOXP4
β-actin
replicate-1
replicate-2
replicate-3

Figure5

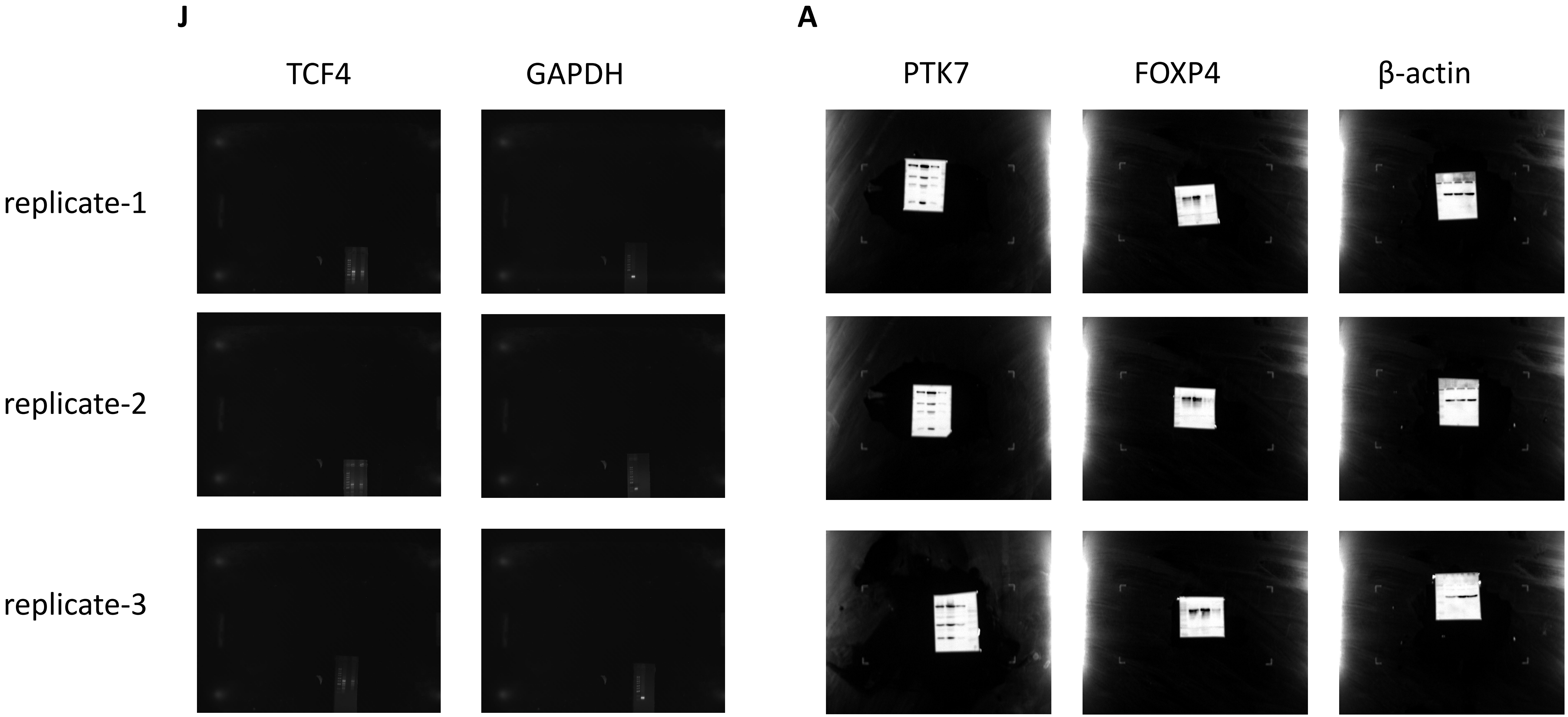
J
A
TCF4
GAPDH
PTK7
FOXP4
β-actin
replicate-1
replicate-2
replicate-3

Figure6

A

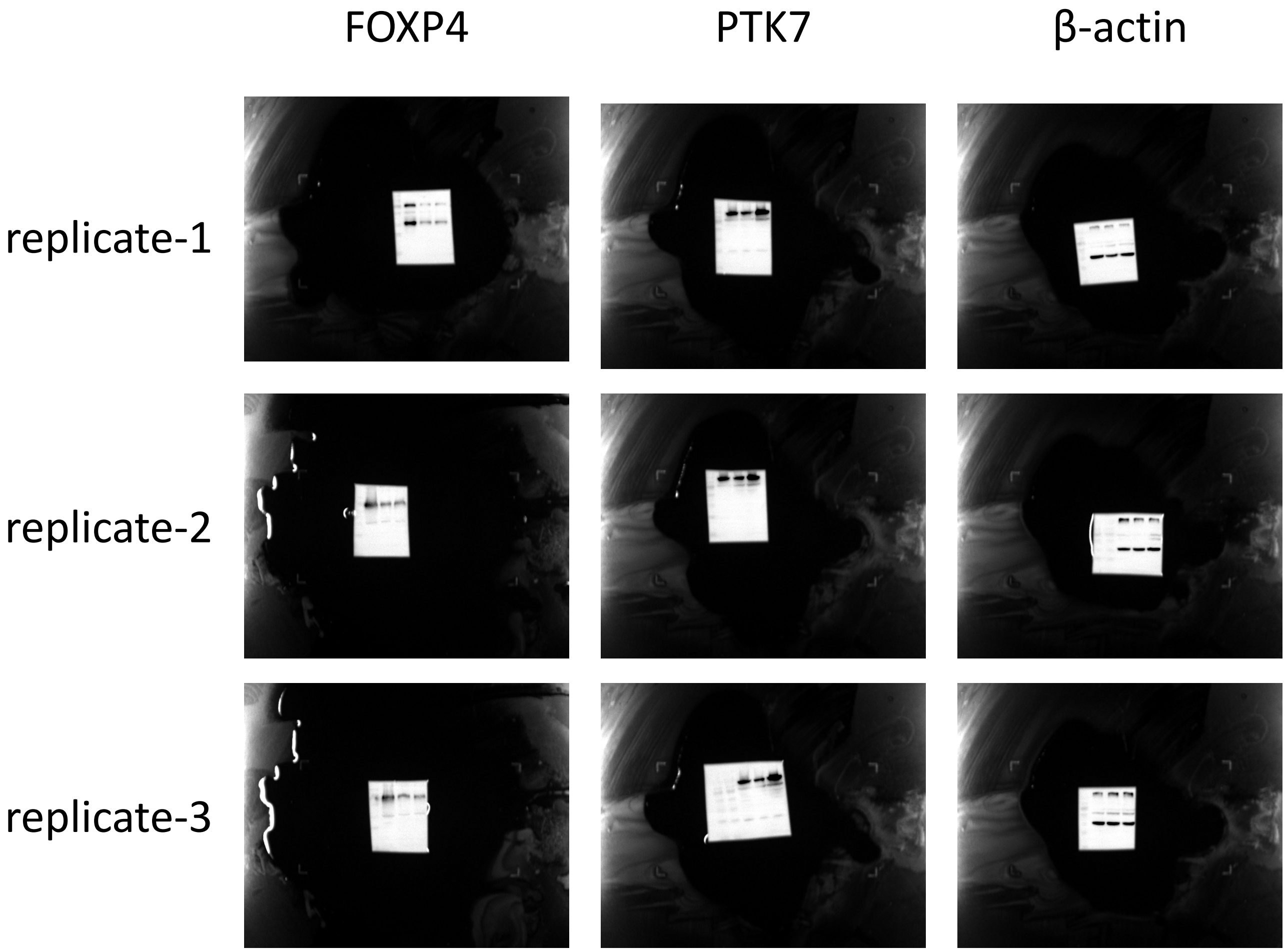

Figure7

A

FOXP4 PTK7 β-actin

replicate-1

replicate-2

replicate-3

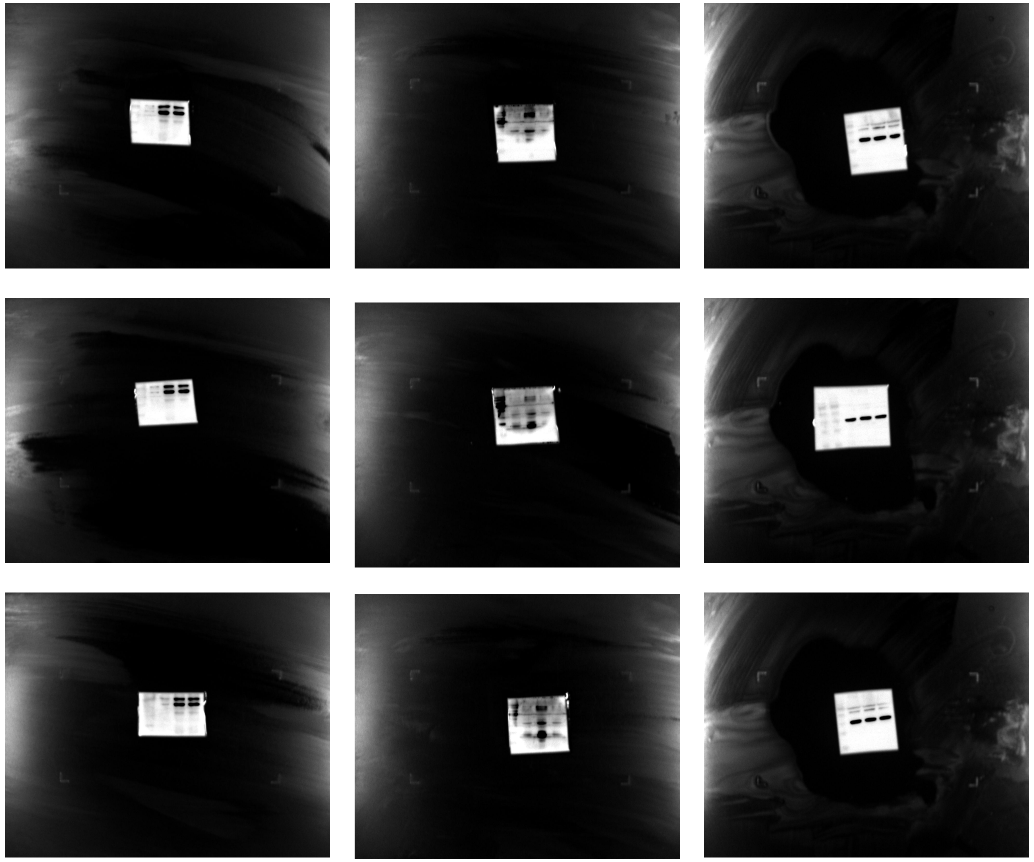

Supplementary Figure 2

A

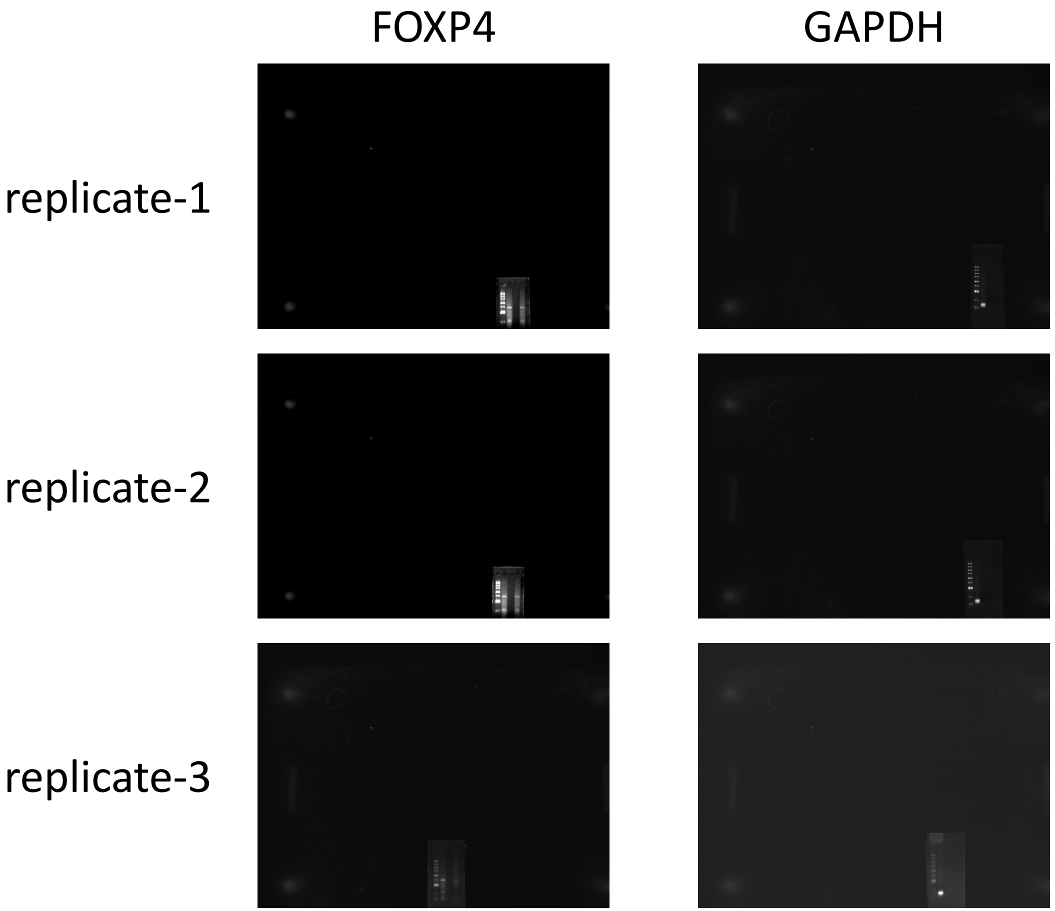

Supplementary Figure 4

A

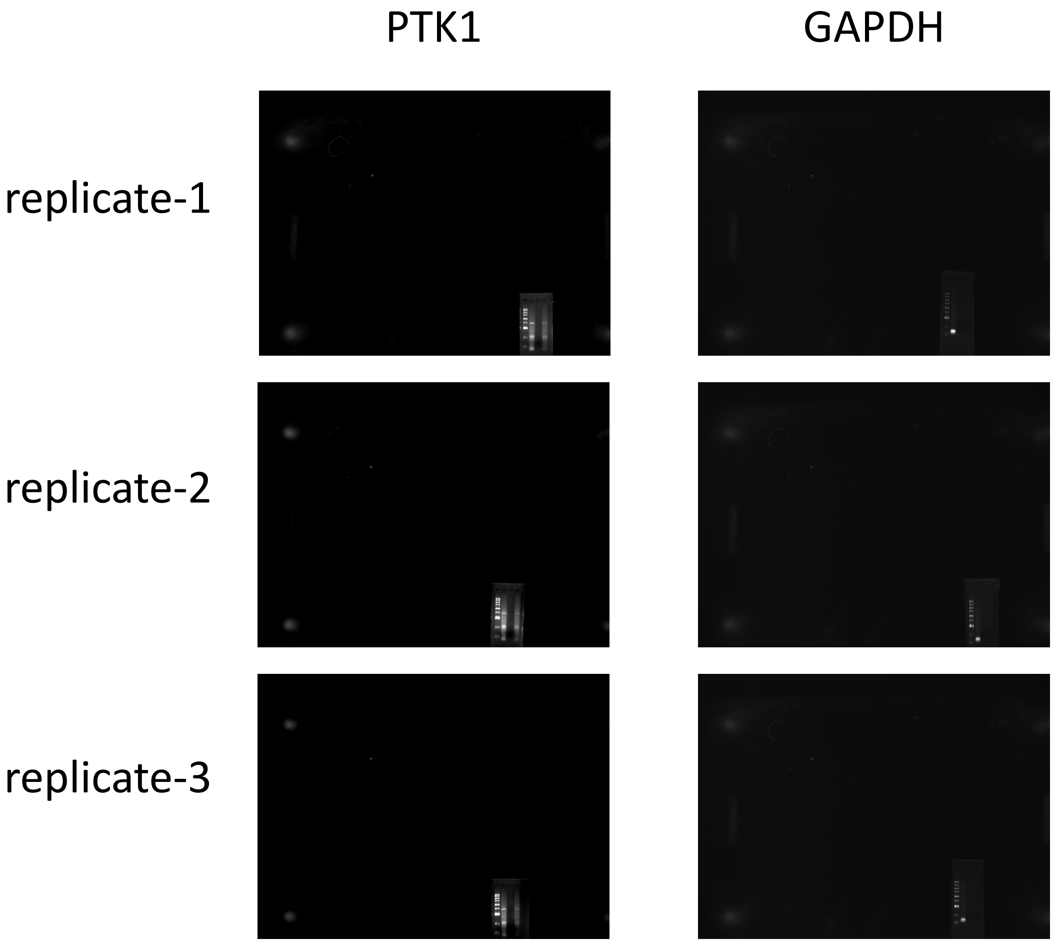

Supplementary Figure 5

A

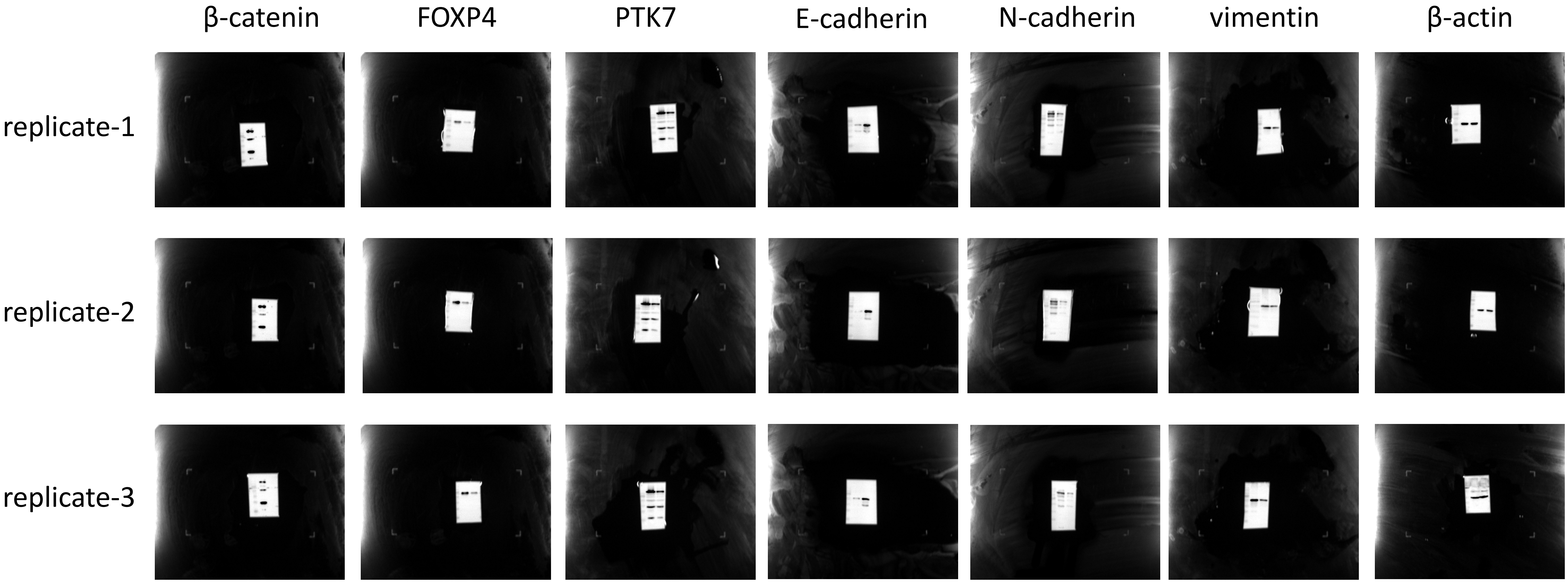

Supplementary Figure 5

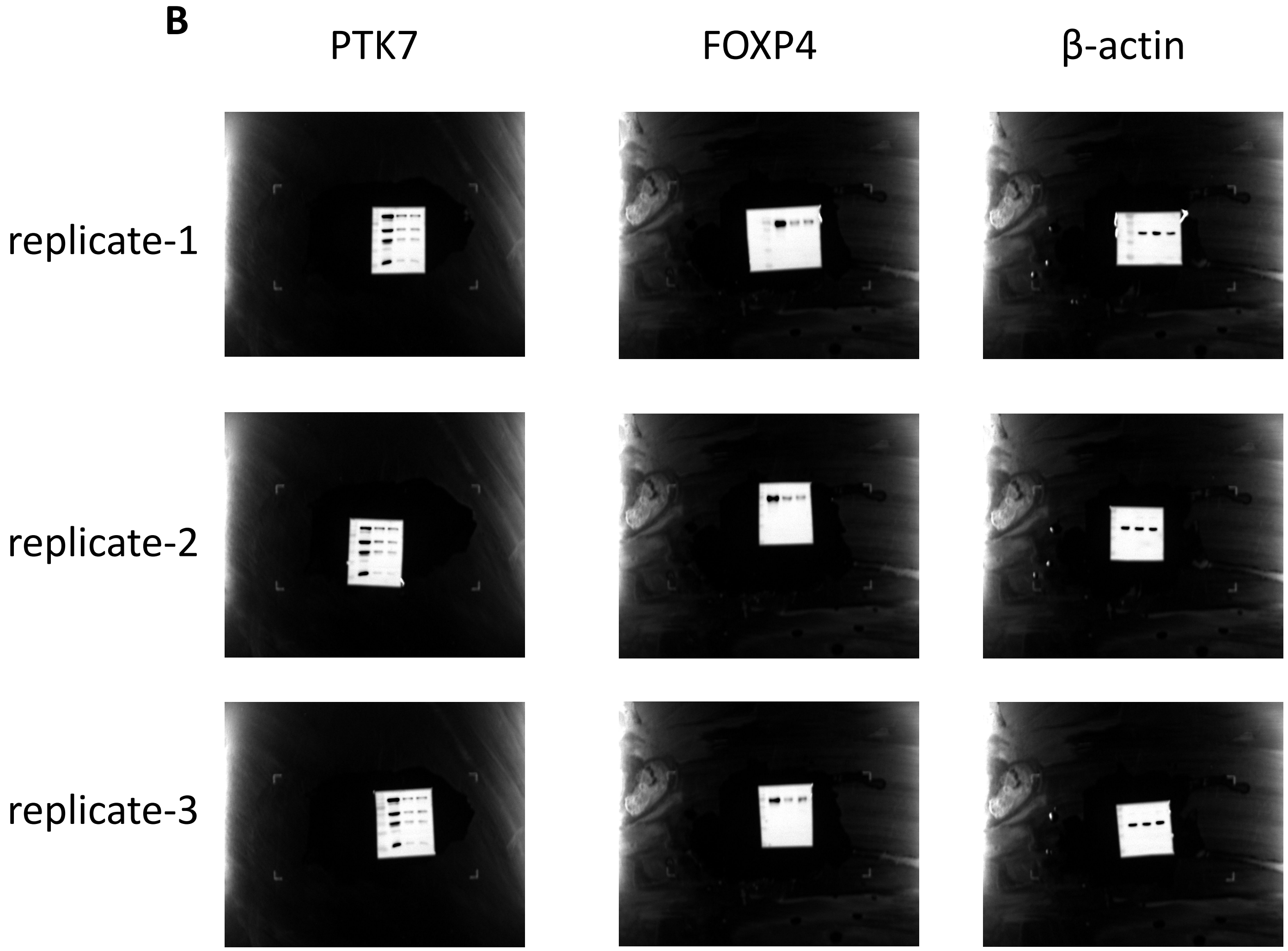

Supplement: Supplementary file 3 — Full and uncropped western blot [file 41419_2024_6713_MOESM3_ESM.pdf]
